# Analysis of genome variants in dwarf soybean lines obtained in F6 derived from cross of normal parents (cultivated and wild soybean)

**DOI:** 10.5808/gi.21024

**Published:** 2021-06-30

**Authors:** Neha Samir Roy, Yong-Wook Ban, Hana Yoo, Rahul Vasudeo Ramekar, Eun Ju Cheong, Nam-Il Park, Jong Kuk Na, Kyong-Cheul Park, Ik-Young Choi

**Affiliations:** 1 Department of Agriculture and Life Industry, Kangwon National University, Chuncheon 24341, Korea; 2 Department of Forest Environmental System, Kangwon National University, Chuncheon 24341, Korea; 3 Department of Plant Science, Gangneung-Wonju National University, Gangneung 25457, Korea; 4 Department of Controlled Agriculture, Kangwon National University, Chuncheon 24341, Korea

**Keywords:** dwarf, RIL population, SNP, soybean, whole genome sequencing, wild type

## Abstract

Plant height is an important component of plant architecture and significantly affects crop breeding practices and yield. We studied DNA variations derived from F5 recombinant inbred lines (RILs) with 96.8% homozygous genotypes. Here, we report DNA variations between the normal and dwarf members of four lines harvested from a single seed parent in an F6 RIL population derived from a cross between *Glycine max* var. Peking and *Glycine soja* IT182936. Whole genome sequencing was carried out, and the DNA variations in the whole genome were compared between the normal and dwarf samples. We found a large number of DNA variations in both the dwarf and semi-dwarf lines, with one single nucleotide polymorphism (SNP) per at least 3.68 kb in the dwarf lines and 1 SNP per 11.13 kb of the whole genome. This value is 2.18 times higher than the expected DNA variation in the F6 population. A total of 186 SNPs and 241 SNPs were discovered in the coding regions of the dwarf lines 1282 and 1303, respectively, and we discovered 33 homogeneous nonsynonymous SNPs that occurred at the same loci in each set of dwarf and normal soybean. Of them, five SNPs were in the same positions between lines 1282 and 1303. Our results provide important information for improving our understanding of the genetics of soybean plant height and crop breeding. These polymorphisms could be useful genetic resources for plant breeders, geneticists, and biologists for future molecular biology and breeding projects.

## Introduction

Soybean is one of the most important leguminous crops worldwide due to its use in human food and oil production. Currently, the United States, Brazil, and Argentina account for more than 80% of the worldwide production of soybean [1]. In Southeast Asian countries, particularly Korea, China, and Japan, soybean is used in multiple life stages as a rich source of protein, and it is considered one of the five major grains [2]. Plant height is an important trait that has a direct impact on yield and lodging resistance. Extremely tall plants can be affected by lodging, which may reduce yield and quality [3]. Dwarfism in crops has played a major role in the “Green Revolution,” in which semi-dwarf varieties were chosen for further cultivation, first in wheat and then in rice [4]. Many studies of plant height inheritance have successfully cloned dwarf genes [5-7]. In soybean, some high-yielding and lodging-resistant dwarf varieties have been developed [8].

The rapid development of next-generation sequencing (NGS) technology and instruments has supported quick and efficient genomics research [9,10]. The key advantage of NGS is that it can produce a large amount of data at low cost, and it is currently being applied to a number of plants [11-14]. Single nucleotide polymorphisms (SNPs) are ubiquitous in genomes and have emerged as a marker of choice, especially in sequenced plants [15,16]. Plants adapt to different environments by various mechanisms, one of which is allelic variation. The identification of these variations is the first step in the in-depth study of the genes and alleles involved in plant evolution and environmental adaptation.

A previously reported study, where the wild soybean *Glycine soja* was sequenced and compared to the *Glycine max* reference, found 2.5 megabases (Mb) of substituted sequences, 4.6 kilobases (kb) of indels, 32.4 Mb of deletions and 8.3 Mb of new sequences in a total of 915.5 Mb of genome sequence [14]. Although a great deal of information is available from whole genome sequencing, resequencing strategies have become an important tool to study allelic variations. There have been studies in other plants, such as rice [17], maize [18], *Arabidopsis* [13], and sorghum [19], as well as resequencing studies in soybean, where both wild and commercial varieties have been analyzed [20-22]. In this study, we compared two inbred lines obtained from a cross between *G. max* var. Peking and *G. soja* IT182936 in the F6 generation. A few lines segregated for a dwarf phenotype and continued to show the same phenotype in the next generation. Resequencing analysis revealed many SNPs and indels in both genic and non-genic regions, which are explained in this study.

## Methods

### Plant materials

Recombinant inbred lines (RILs) were developed from a cross between *G. max* var. Peking and *G. soja* IT182936. The soybeans used in this experiment were harvested in Chuncheon city (Gangwon-do, South Korea). All the plants were grown in field condition. Two RILs exhibiting normal and dwarf phenotype from F6 generation were selected for whole genome variant analysis and designated 1282NF6 and 1303NF6 for normal plants and 1282DF6 and 1303DF6 for dwarf plants. Two more RILs, 1214 and 1290, exhibiting semi-dwarf phenotypes, were also selected for comparison with the dwarf lines. Three leaves were collected from each plant before flowering, frozen immediately in liquid nitrogen and stored at ‒80℃.

### DNA isolation and Illumina sequencing

Genomic DNA was isolated from the leaf tissues using the modified CTAB method [23]. DNA purification was carried out using QIAquick Purification Kit (28104, Qiagen, Beijing, China). Adaptor ligation and DNA clustering preparations were done by Solexa sequencing using Illumina HiSeq 4000 sequencing platform according to the manufactures' protocol by the National Instrumentation Center for Environmental Management (NICEM) at Seoul National University. The sequencing libraries were prepared by random fragmentation of the DNA sample, followed by 5' and 3' adaptor ligation. Low-quality reads (ratio of reads that have phred quality score of <20), reads with adaptor sequences, and duplicated reads were eliminated. The remaining high-quality data were used for mapping. We used the published genome sequence of *G. max* version 1 as a reference [24].

### Identification of DNA variations in normal and dwarf lines

The raw Illumina sequencing data were filtered and compared to characterize the genotype of normal and dwarf samples using the Bowtie2 (v2.3.4.3) aligner [25]. The SNPs were qualified by GATK (version 2.3.9 Lite) [26] and biallelic filtering. GATK filtering was performed with the options MQ0 ≥ 4 && ((MQ0/(1.0*DP)) > 0.1), QUAL < 30, QD < 5.0, and FS > 200.0 [27]. The biological function of each SNP and indel locus were identified using SnpEff software [28]. Paired-end reads were mapped against the TAIR10 reference genome sequence [29]. The DNA variations common to the two sets of dwarf lines were discovered by comparing the SNPs discovered at the sample loci between the normal and dwarf samples.

## Results

### In silico mapping of resequencing reads to reference and variant calls

**Fig. 1.**
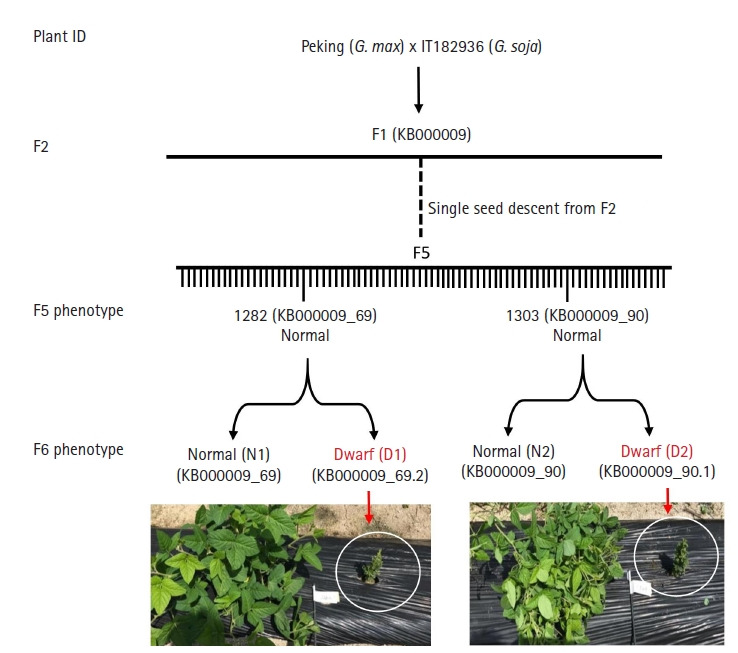
Schematic representation of dwarf recombinant inbred lines (RIL) development. *Glycine max* var. Peking and *G. soja* var. IT182936 were crossed to develop RIL lines. From F2 few lines exhibited dwarf phenotype and continued to appear dwarf in their successive generation. Two such lines from the F6 generation were chosen for analysis.

The parental lines (*G. max* var. Peking and *G. soja* IT182936) did not exhibit dwarfism, but few plants in F3 generation appeared dwarf (Fig. 1). Although F3 dwarf lines didn’t produce any seeds, there were five dwarf lines in next generation (F3). Two out of five produced seeds and continued to produce dwarf phenotype in their next generation. Two of such samples, 1282 normal and dwarf (labeled 1282NF6 and 1282DF6) and 1303 normal and dwarf (labeled 1303NF6 and 1303DF6) from F6 generation were chosen and used for genomics variation analysis using Illumina sequencing method. Additional two samples that exhibited semi-dwarf phenotype were also used to analysis viz. (labeled 1214NF6 and 1214DF6 and 1290NF6 and 1290DF6). The total number of reads obtained for 1282NF6 was 159,196,424, which accounted for 24 G bps. The GC content was 35.65% with a Q20 value of 94.82%. Likewise, the total reads for 1282DF6 were 172,215,288, accounting for 26 Gbps, and in line 1303, the numbers of total reads for the normal and dwarf samples were 150,712,004 and 139,484,320, accounting for 22 Gbps and 21 Gbps, respectively (Supplementary Table 1). The GC content for all the lines was above 35%, and the Q20 average percentage was above 94%. More than 93%, on average, was mapped to the reference genome. There were 5,597,100 variant calls in both genotypes (Table 1). The raw data was deposited in NCBI SRA database with an accession number PRJNA665611.

To estimate the number of variants that can be obtained in a hybrid of *G. max* and *G. soja* we need to know the total number of SNPs and indels in them. G. Ramakrishna et al. identified a total 77,339 SNPs and 451,522 indels in *G. max* whereas 215,932 SNPs and 697,295 indels in *G. soja*, with comparison to reference post-filtering [30]. Among them, the number of common variants for both species was 10,873 SNPs and 80,078 indels. So if we exclude the common SNPs and indels from both species, we would be left with 282,398 SNPs and 1,069,739 indels making the total count of variations into 1,351,137. As per the Mendelian genetics, the cross between *G. max* and *G. soja* will distribute the variants into half from each parent to its offspring F1 into 1:2:1 ratio of both parents, then subsequently to the next F2 generation and so on [31].

**Fig. 2.**
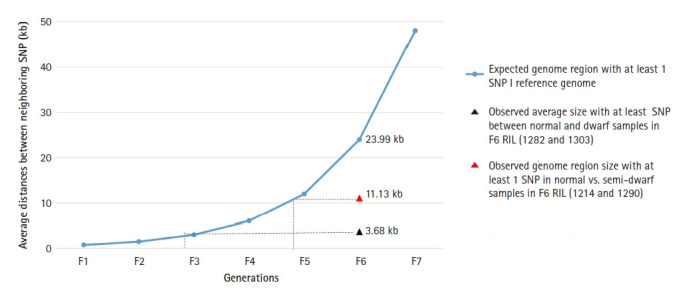
Single nucleotide polymorphism (SNP) per kb of gene length in dwarf soybean lines. The frequency of SNP in dwarf lines was observed significantly higher than expected. Dwarf lines exhibited SNP on every 3.68 kb of genome whereas the expected length of the genome should be 23.99 kb. RIL, recombinant inbred lines.

With respect to that, theoretically the distribution of SNP in resulting cross should be 1 SNP per 0.748 kb in F1 generation (genome size 1,013,200 kb/total variation 1,351,137) [31]. Therefore in F2 generation the distribution is 1 SNP per 1.49 kb and so on. Consequently in theory in F6 generation there should be 1 SNP per 23.99 kb. From the filtered SNPs, we obtained average 217,764 and 57,403 homozygous SNP in dwarf lines and semi-dwarf lines of F6 generation (Table 2). Implying there is 1 SNP per 3.68 kb region of dwarf lines and 11.13 kb region of semi-dwarf lines (Fig. 2).

### Distribution of SNPs in coding regions of the reference genome

**Fig. 3.**
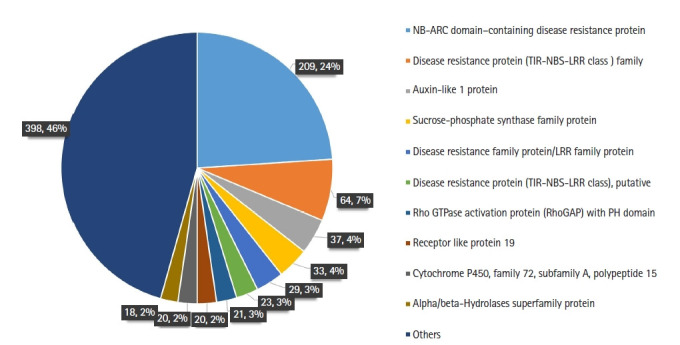
Distribution of missense single nucleotide polymorphisms (SNPs) in the soybean genome in genic regions. NB-ARC, nucleotide-binding APAF-1 R proteins and CED-4; TIR-NBS-LRR, toll interleukin 1 receptor nucleotide-binding site leucine-rich repeat resistance proteins.

The genes that directly affect the growth of plants viz. plant defense, phytohormones, and photosynthesis were considered while comparing the SNPs in dwarf and normal lines. Furthermore we focused exclusively on missense SNPs on coding regions as they may cause base changes in protein sequence and alter the gene function. We observed 503 and 485 SNPs among dwarf and normal of 1,282 and 1,303, respectively, out of which 98 were common to both genotypes when comparison to reference genome. The highest number of SNPs was observed in NB-ARC (nucleotide-binding APAF-1 R proteins and CED-4) domain–containing disease resistance protein (75%), followed by disease resistance protein (TIR-NBS-LRR [toll interleukin 1 receptor nucleotide-binding site leucine-rich repeat resistance proteins] class) family (35%) and auxin-like 1 protein (27%) (Fig 3, Supplementary Table 2). The distribution was highest on chromosome (Chr) 18, followed by Chr 16, for both genotypes. When considering the individual nonsynonymous SNP distribution, we observed that Chr 16 had the maximum number of SNPs in both 1282 (74) and 1303 (67), followed by Chr 7, with 58 and 63 in 1282 and 1303, respectively (Supplementary Table 3). We discovered a total of 33 homogeneous nonsynonymous SNPs that occurred at the same loci in each set of dwarf and normal soybean derived from normal soybean. We then identified the homogeneous SNPs across all the dwarf samples, with the highest representation of SNPs on Chr 16 and Chr 7 in both genotypes. SNPs that were common to both genotypes were highest on Chr 4, followed by Chr 7 and Chr 15 (Supplementary Table 3).

There were three nonsense SNPs found in both genotypes: one was on Chr 2, another was on Chr 13, and the last was on Chr 20; the SNP from Chr 20 was homogeneous in the dwarf lines (Supplementary Table 4). The three nonsense SNPs obtained in the genic regions included proteins with the gene functions single-stranded DNA (ssDNA)-binding transcriptional regulator, UDP-glycosyltransferase protein (UGT) and PIF1 helicase. Twenty frameshift SNPs were common to both genotypes (Supplementary Table 4). The frameshift mutations observed in the genic regions of the normal and dwarf individuals are shown in Supplementary Table 3. Out of 20 mutated genes, five were leucine-rich receptor-like protein family genes; three were proteins of unknown function (DUF647); two were NADH-ubiquinone/plastoquinone oxidoreductase chain 4L, 2 cytochrome P450, family 78, subfamily A, polypeptide 5; and one each was MUTS homolog 6, unknown protein and RNA helicase-like 8.

### Distribution of SNPs among normal and dwarf plants

When we considered the SNP among normal and dwarf lines and not with the reference, 426 SNPs were obtained, among which five were common to both samples (1282 and 1303) (Table 3). The SNPs were distributed on all chromosomes except 3, 4, 5, 16, and 17. Sample 1282 had the highest number of SNPs on Chr 7 (36), followed by Chr 1 (29) and Chr 13 (27). Likewise, sample 1303 had the highest number of SNPs on Chr 7 (71), followed by Chr 2 (54) and Chr 13 (28). The gene functions of the SNPs that were common to both normal and dwarf samples in both lines (1282 and 1303) included matrixin family protein, zinc finger protein, transcription factor and cytochrome P450 protein polypeptide (Table 4).

## Discussion

RIL lines were developed from *G. max* and *G. soja* and none of the parent exhibited dwarf phenotype. From F3 generation we obtained few lines that exhibited dwarf phenotype which continued to produce dwarf lines in subsequent generation. We chose two lines from F6 that had both phenotypes (dwarf and normal) to survey the variance among them. Genome variant analysis was performed in such lines from F6 generation exhibiting dwarf and semi-dwarf phenotype. We observed that the dwarf lines in this study had a higher number of SNPs and indels than the semi-dwarf lines (Table 2). This implies that higher number of variations could cause changes in gene function causing dwarf phenotype. We obtained 5M of total variation in our data (Table 1). The number although is much smaller than that obtained in a recent study in *G. soja* [32] which was more than 15M SNPs and 14M indels. The reason could be due to the fact that they have used more than 26 accessions to determine variants whereas we have used only one Wm82 genome to look for the differences. Initially, we compared the number of SNPs with respect to reference G max genome where we observed a smaller number of SNPs in semi-dwarf lines and a higher number in dwarf lines. The homozygosity in F5 and F6 RILs are 94% and 97%, respectively [31]. This can explain the higher numbers of variants which may also impact the phenotype. Although the number of SNPs observed in the F6 populations in our study was greater between the dwarf and normal plants (1 SNP per 3.68 kbp for dwarf and 11.13 kb for semi-dwarf plants) (Fig. 2). This is much higher than the normal SNP frequency in the F6 generation, which is 1 per 23.99 kbp [33].

Natural variations in the genome, such as SNPs in coding regions, can alter amino acid sequences and modify the post-translation products, which may affect gene function [34,35]. Notably, disease resistance protein genes exhibited a high number of variants in our study. Similar studies have shown that changes in protein function (gain or loss of function) may contribute to dwarf phenotypes. Dwarfism due to a gain-of-function mutation in a TIR-NB-LRR protein was reported in *Arabidopsis* as one of the mechanisms underlying enhanced disease resistance [36]. Moreover, changes in these proteins cause autoimmunity in plants, and one of the useful features of autoimmune mutants is their dwarf phenotype [37]. Temperature and humidity are known to play important roles in dwarfism, although the exact mechanism is still unknown [38-42]. The RILs in our study were all grown in the same environmental conditions with the same temperature and humidity. This suggests that the dwarf phenotypes observed in our study were not due to temperature or humidity.

The SNPs were mostly in the NB-ARC domain‒containing disease resistance protein, followed by disease resistance protein and auxin-like protein (Fig. 3). In *Arabidopsis*, NB-ARC mutants induced autoimmunity in plants, of which dwarfism is one of the typical phenotypes [37]. Common SNPs occurring in gene coding regions could affect the phenotype, whether or not they are combined with other genes. There is not much known about the matrixin family protein in plants, but the members of the matrix metalloproteinase family are thought to be involved in remodeling of the extracellular matrix during plant growth and development [43]. Thus, an SNP in a gene encoding a member of this protein family might have caused an alteration in protein function leading to dwarfing. Mutations in the transcription factor jumonji (jmjC) have been reported to complement plant growth defects and expression changes [44]. Overexpression of TTF-type zinc finger protein in *Arabidopsis* resulted in divergent physiological and metabolic phenotypes, some of which were significant for improved plant performance [45]. The SNPs occurring in these vital genes may have contributed to the dwarf phenotypes found in the RILs.

There were nonsense SNPs in three loci: ssDNA-binding transcriptional regulator, UGT superfamily protein, and PIF1 helicase. ssDNA-binding transcriptional regulators are known to function as positive and negative regulators in leaf senescence [46]. UGTs also act as major contributors to plant growth, including development, disease resistance, and interaction with the environment, by interacting with various substrates, such as flavonoids, terpenes, auxins, cytokinin, and many others [47]. PIF1 helicases are enzymes that are essential in DNA replication, repair, and recombination in all organisms. Likewise, frameshift mutations were found in genes with important functions, such as leucine-rich receptor-like protein family, protein of unknown function (DUF647),NADH-ubiquinone/plastoquinone oxidoreductase chain 4L, cytochrome P450 family 78-subfamily A polypeptide 5, MUTS homolog 6, unknown protein and RNA helicase-like 8. All these proteins are major regulators and contributors to plant growth and development.

The SNPs obtained in our study (missense, nonsense SNP, and frameshift mutations) were in vital genes but may or may not have impacted plant growth. We were unable to derive any particular conclusion about the dwarf phenotype based on our results, but this study will provide a basis to analyze further and evaluate which SNPs affect plant growth type. The identification of functional SNPs in genes and analysis of their effects on phenotype may lead to a better understanding of their impacts on gene function and thus support varietal improvement.

## Figures and Tables

**Table 1.** Variant calling statistics as compared to reference genome

Raw variants (SNP + INDEL)	SNP		INDEL	
	Raw variants	Filtered	Raw variants	Filtered
5,597,100	4,645,717	4,108,601	944,240	904,602

SNP, single nucleotide polymorphism.

**Table 2.** Number of candidate variants after filtering with reference genome

Sample	Different between normal and dwarf (SNP)	Homozygous in dwarfism sample (SNP)	Different between normal and dwarf (INDEL)	Homozygous in dwarfism sample (INDEL)
1282	458,209	182,497	108,277	51,622
1303	337,001	253,032	100,267	63,184
1214	116,136	65,599	55,767	30,225
1290	111,052	57,098	56,000	29,152

SNP, single nucleotide polymorphism.

**Table 3.** Chromosome wise distribution of SNP among normal and dwarf lines

Chromosome No.	1282 Normal and dwar	1303 Normal and dwarf	1282 + 1303 Common SNPs
1	29	18	2
2	7	54	0
6	3	0	0
7	36	71	0
8	23	17	0
9	11	11	0
10	17	8	0
11	4	6	0
12	1	2	0
13	27	28	0
14	3	1	0
15	22	0	0
19	13	11	2
20	3	1	1
Total	186	241	5

SNP, single nucleotide polymorphism.

**Table 4.** Common SNP among normal and dwarf lines

Gene	Chromosome No.	1282NF6	1282DF6	1303NF6	1303DF6	TAIR TOP hit function
GLYMA01G04370	1	G/G	A/G	G/G	A/G	Matrixin family protein
GLYMA01G06671	1	C/C	T/T	T/T	C/C	TTF-type zinc finger protein with HAT dimerization domain
GLYMA19G14700	19	A/G	A/A	A/G	A/A	Transcription factor jumonji (jmjC) domain‒containing protein
GLYMA19G14700	19	G/G	G/A	G/A	A/A	Transcription factor jumonji (jmjC) domain‒containing protein
GLYMA1057S00200	20	T/C	T/T	T/T	T/C	Cytochrome P450, family 76, subfamily C, polypeptide 4

SNP, single nucleotide polymorphism.

## Supplementary Materials

Supplemental Table 1. Raw data statistics in dwarf soybean lines

Supplemental Table 2. Distribution of missense SNP in soybean genome in both samples (1282 and 1303

Supplemental Table 3. Distribution of missense SNP when compared with reference genome

Supplemental Table 4. Nonsense and frameshift in both samples as compared to reference genome
