# Supplementary material for: Analysis of genome variants in dwarf soybean lines obtained in F6 derived from cross of normal parents (cultivated and wild soybean)
Source: Genomics Inform. 2021 Jun 30;19(2):e19. doi: 10.5808/gi.21024 (PMC8261272; doi:10.5808/gi.21024)
Supplement: Supplemental Table 1. — Raw data statistics in dwarf soybean lines [file gi-21024suppl1.pdf]

Supplementary Table 1. Raw data statistics in dwarf soybean lines

Sample ID	Total read bases (bp)	Total reads	GC (%)	Q20	Reads mapped	Percentage mapped
1282NF6	24,038,660,024	159,196,424	36.17	94.82	147,556,451	94.65
1282DF6	26,004,508,488	172,215,288	36.46	94.23	158,358,368	92.72
1303NF6	22,757,512,604	150,712,004	35.62	94.972	141,181,553	94.47
1303DF6	21,017,028,922	139,185,622	36.19	95.16	128,813,719	93.32
1214NF6	24,755,898,850	163,946,350	35.64	94.72	153,842,932	94.65
1214DF6	23,739,433,592	157,214,792	36.17	94.56	145,987,368	93.63
1290NF6	20,911,132,320	138,484,320	35.77	95.14	129,510,201	94.47
1290DF6	22,240,139,492	147,285,692	35.06	95.28	137,687,083	94.26

Sample ID, sample name; Total read bases, total number of bases sequenced; Total reads, total number of reads. In Illumina paired-end sequencing, read1 and read2 were added together; GC (%), GC content; Q20 (%), ratio of reads with a Phred quality score of over 20.
