# Supplementary material for: Analysis of genome variants in dwarf soybean lines obtained in F6 derived from cross of normal parents (cultivated and wild soybean)
Source: Genomics Inform. 2021 Jun 30;19(2):e19. doi: 10.5808/gi.21024 (PMC8261272; doi:10.5808/gi.21024)
Supplement: Supplemental Table 2. — Distribution of missense SNP in soybean genome in both samples (1282 and 1303 [file gi-21024suppl2.pdf]

Supplementary Table 2. Distribution of missense SNP in soybean genome in both samples (1282 and 1303)

Gene	Transcript	Chrom	Position	Ref	Alt	DNA	Protein	TAIR TOP hit	TAIR TOP hit function
GLYMA01G04370	GLYMA01G04370.1	1	3910902	A	G	58A>G	Lys20Glu	AT1G59970	Matrixin family protein
GLYMA01G04370	GLYMA01G04370.1	1	3910914	T	G	70T>G	Ser24Ala	AT1G59970	Matrixin family protein
GLYMA01G33326	GLYMA01G33326.1	1	45317579	A	G	482T>C	Val161Ala	ATMG01320	NADH dehydrogenase 2B
GLYMA01G06671	GLYMA01G06671.1	1	7053682	G	A	358G>A	Glu120Lys	AT1G19260	TTF-type zinc finger protein with HAT dimerisation domain
GLYMA01G06671	GLYMA01G06671.1	1	7053719	C	T	395C>T	Ala132Val	AT1G19260	TTF-type zinc finger protein with HAT dimerisation domain
GLYMA01G06671	GLYMA01G06671.1	1	7053921	T	G	597T>G	Ile199Met	AT1G19260	TTF-type zinc finger protein with HAT dimerisation domain
GLYMA01G06671	GLYMA01G06671.1	1	7054079	G	A	755G>A	Arg252His	AT1G19260	TTF-type zinc finger protein with HAT dimerisation domain
GLYMA01G33326	GLYMA01G33326.1	1	45317688	T	C	373A>G	Ile125Val	ATMG01320	NADH dehydrogenase 2B
GLYMA01G33326	GLYMA01G33326.1	1	45317708	T	A	353A>T	Tyr118Phe	ATMG01320	NADH dehydrogenase 2B
GLYMA01G25758	GLYMA01G25758.1	1	33877931	A	G	200A>G	His67Arg	AT3G51690	PIF1 helicase
GLYMA01G25758	GLYMA01G25758.1	1	33878197	C	A	466C>A	Pro156Thr	AT3G51690	PIF1 helicase
GLYMA01G09631	GLYMA01G09631.1	1	11934602	A	G	80A>G	His27Arg	AT1G03280	Transcription factor TFIIE, alpha subunit
GLYMA01G09631	GLYMA01G09631.1	1	11934652	A	G	130A>G	Thr44Ala	AT1G03280	Transcription factor TFIIE, alpha subunit
GLYMA01G25758	GLYMA01G25758.1	1	33878212	C	T	481C>T	His161Tyr	AT3G51690	PIF1 helicase
GLYMA01G25758	GLYMA01G25758.1	1	33878018	T	A	287T>A	Leu96Gln	AT3G51690	PIF1 helicase
GLYMA01G29580	GLYMA01G29580.2	1	39900713	T	A	1781T>A	Ile594Asn	AT1G45616	Receptor like protein 6
GLYMA01G06671	GLYMA01G06671.1	1	7053471	T	C	182T>C	Val61Ala	AT1G19260	TTF-type zinc finger protein with HAT dimerisation domain
GLYMA01G25758	GLYMA01G25758.1	1	33878489	A	C	704A>C	His235Pro	AT3G51690	PIF1 helicase
GLYMA01G26900	GLYMA01G26900.2	1	35720214	G	A	241G>A	Val81Ile	AT2G37960	N/A
GLYMA01G29580	GLYMA01G29580.2	1	39900533	T	A	1601T>A	Ile534Asn	AT1G45616	Receptor like protein 6

GLYMA01G29580	GLYMA01G29580.2	1	39900576	C	G	1644C>G	Ile548Met	AT1G45616	Receptor like protein 6
GLYMA01G06671	GLYMA01G06671.1	1	7055316	T	C	1925T>C	Leu642Pro	AT1G19260	TTF-type zinc finger protein with HAT dimerisation domain
GLYMA01G29580	GLYMA01G29580.2	1	39900755	C	A	1823C>A	Pro608Gln	AT1G45616	Receptor like protein 6
GLYMA01G06671	GLYMA01G06671.1	1	7055379	G	A	1988G>A	Arg663His	AT1G19260	TTF-type zinc finger protein with HAT dimerisation domain
GLYMA01G06671	GLYMA01G06671.1	1	7055463	A	G	2072A>G	Gln691Arg	AT1G19260	TTF-type zinc finger protein with HAT dimerisation domain
GLYMA01G30560	GLYMA01G30560.1	1	41232951	C	T	912G>A	Met304Ile	N/A	N/A
GLYMA01G06671	GLYMA01G06671.1	1	7055479	T	G	2088T>G	Phe696Leu	AT1G19260	TTF-type zinc finger protein with HAT dimerisation domain
GLYMA01G30560	GLYMA01G30560.1	1	41232665	G	C	1124C>G	Thr375Arg	N/A	N/A
GLYMA01G30560	GLYMA01G30560.1	1	41233271	A	G	622T>C	Cys208Arg	N/A	N/A
GLYMA01G30560	GLYMA01G30560.1	1	41232826	A	G	992T>C	Ile331Thr	N/A	N/A
GLYMA01G30560	GLYMA01G30560.1	1	41233811	C	T	229G>A	Glu77Lys	N/A	N/A
GLYMA01G30560	GLYMA01G30560.1	1	41233069	G	C	794C>G	Ser265Cys	N/A	N/A
GLYMA01G30560	GLYMA01G30560.1	1	41233093	T	C	770A>G	Asp257Gly	N/A	N/A
GLYMA01G33326	GLYMA01G33326.1	1	45317645	A	G	416T>C	Leu139Ser	ATMG01320	NADH dehydrogenase 2B
GLYMA01G33326	GLYMA01G33326.1	1	45317663	T	C	398A>G	Asn133Ser	ATMG01320	NADH dehydrogenase 2B
GLYMA01G30560	GLYMA01G30560.1	1	41233381	T	C	512A>G	His171Arg	N/A	N/A
GLYMA01G30560	GLYMA01G30560.1	1	41234160	G	A	95C>T	Ser32Leu	N/A	N/A
GLYMA01G37406	GLYMA01G37406.1	1	49768140	A	T	616A>T	Met206Leu	AT2G18090	N/A
GLYMA01G37406	GLYMA01G37406.1	1	49768141	T	C	617T>C	Met206Thr	AT2G18090	N/A
GLYMA01G37406	GLYMA01G37406.1	1	49769179	G	T	1531G>T	Val511Leu	AT2G18090	N/A
GLYMA01G37406	GLYMA01G37406.2	1	49768140	A	T	616A>T	Met206Leu	AT2G18090	N/A
GLYMA01G37406	GLYMA01G37406.2	1	49768141	T	C	617T>C	Met206Thr	AT2G18090	N/A
GLYMA01G37406	GLYMA01G37406.2	1	49769179	G	T	1531G>T	Val511Leu	AT2G18090	N/A
GLYMA02G06980	GLYMA02G06980.4	2	5610115	G	T	1627G>T	Asp543Tyr	AT3G50430	N/A
GLYMA02G06980	GLYMA02G06980.4	2	5610151	G	T	1663G>T	Asp555Tyr	AT3G50430	N/A

GLYMA02G06980	GLYMA02G06980.4	2	5610157	G	A	1669G>A	Gly557Arg	AT3G50430	N/A
GLYMA02G06980	GLYMA02G06980.5	2	5610115	G	T	1627G>T	Asp543Tyr	AT3G50430	N/A
GLYMA02G06980	GLYMA02G06980.5	2	5610151	G	T	1663G>T	Asp555Tyr	AT3G50430	N/A
GLYMA02G06980	GLYMA02G06980.5	2	5610157	G	A	1669G>A	Gly557Arg	AT3G50430	N/A
GLYMA02G06980	GLYMA02G06980.6	2	5610115	G	T	1540G>T	Asp514Tyr	AT3G50430	N/A
GLYMA02G06980	GLYMA02G06980.6	2	5610151	G	T	1576G>T	Asp526Tyr	AT3G50430	N/A
GLYMA02G06980	GLYMA02G06980.6	2	5610157	G	A	1582G>A	Gly528Arg	AT3G50430	N/A
GLYMA02G12490	GLYMA02G12490.1	2	10772310	G	C	958C>G	Pro320Ala	AT1G13960	WRKY DNA-binding protein 4
GLYMA02G12490	GLYMA02G12490.1	2	10772857	C	G	788G>C	Ser263Thr	AT1G13960	WRKY DNA-binding protein 4
GLYMA02G13230	GLYMA02G13230.2	2	11484333	G	A	62G>A	Ser21Asn	AT1G75310	Auxin-like 1 protein
GLYMA02G13230	GLYMA02G13230.2	2	11484377	A	G	106A>G	Ile36Val	AT1G75310	Auxin-like 1 protein
GLYMA02G13230	GLYMA02G13230.2	2	11484413	G	A	142G>A	Asp48Asn	AT1G75310	Auxin-like 1 protein
GLYMA02G13230	GLYMA02G13230.2	2	11484488	C	G	217C>G	Gln73Glu	AT1G75310	Auxin-like 1 protein
GLYMA02G13230	GLYMA02G13230.2	2	11484506	G	C	235G>C	Asp79His	AT1G75310	Auxin-like 1 protein
GLYMA02G13230	GLYMA02G13230.2	2	11484545	A	G	274A>G	Ser92Gly	AT1G75310	Auxin-like 1 protein
GLYMA02G13230	GLYMA02G13230.2	2	11486048	T	G	760T>G	Ser254Ala	AT1G75310	Auxin-like 1 protein
GLYMA02G13230	GLYMA02G13230.2	2	11486625	T	C	1337T>C	Val446Ala	AT1G75310	Auxin-like 1 protein
GLYMA02G13230	GLYMA02G13230.2	2	11487068	A	G	1780A>G	Thr594Ala	AT1G75310	Auxin-like 1 protein
GLYMA02G13230	GLYMA02G13230.2	2	11487115	T	G	1827T>G	Asp609Glu	AT1G75310	Auxin-like 1 protein
GLYMA02G13230	GLYMA02G13230.2	2	11487128	C	A	1840C>A	Gln614Lys	AT1G75310	Auxin-like 1 protein
GLYMA02G13230	GLYMA02G13230.2	2	11487132	C	T	1844C>T	Thr615Ile	AT1G75310	Auxin-like 1 protein
GLYMA02G13230	GLYMA02G13230.2	2	11487255	C	A	1967C>A	Ala656Asp	AT1G75310	Auxin-like 1 protein
GLYMA02G13230	GLYMA02G13230.2	2	11487830	G	A	2542G>A	Val848Ile	AT1G75310	Auxin-like 1 protein
GLYMA02G13230	GLYMA02G13230.2	2	11488113	A	C	2825A>C	Asn942Thr	AT1G75310	Auxin-like 1 protein
GLYMA02G13230	GLYMA02G13230.2	2	11488158	T	C	2870T>C	Phe957Ser	AT1G75310	Auxin-like 1 protein
GLYMA02G13230	GLYMA02G13230.2	2	11488242	T	C	2954T>C	Leu985Pro	AT1G75310	Auxin-like 1 protein
GLYMA02G13230	GLYMA02G13230.2	2	11489163	C	G	3670C>G	His1224Asp	AT1G75310	Auxin-like 1 protein
GLYMA02G13230	GLYMA02G13230.3	2	11484333	G	A	62G>A	Ser21Asn	AT1G75310	Auxin-like 1 protein
GLYMA02G13230	GLYMA02G13230.3	2	11484377	A	G	106A>G	Ile36Val	AT1G75310	Auxin-like 1 protein

GLYMA02G13230	GLYMA02G13230.3	2	11484413	G	A	142G>A	Asp48Asn	AT1G75310	Auxin-like 1 protein
GLYMA02G13230	GLYMA02G13230.3	2	11484488	C	G	217C>G	Gln73Glu	AT1G75310	Auxin-like 1 protein
GLYMA02G13230	GLYMA02G13230.3	2	11484506	G	C	235G>C	Asp79His	AT1G75310	Auxin-like 1 protein
GLYMA02G13230	GLYMA02G13230.3	2	11484545	A	G	274A>G	Ser92Gly	AT1G75310	Auxin-like 1 protein
GLYMA02G13230	GLYMA02G13230.3	2	11486048	T	G	760T>G	Ser254Ala	AT1G75310	Auxin-like 1 protein
GLYMA02G13230	GLYMA02G13230.3	2	11486625	T	C	1337T>C	Val446Ala	AT1G75310	Auxin-like 1 protein
GLYMA02G13230	GLYMA02G13230.3	2	11487068	A	G	1780A>G	Thr594Ala	AT1G75310	Auxin-like 1 protein
GLYMA02G13230	GLYMA02G13230.3	2	11487115	T	G	1827T>G	Asp609Glu	AT1G75310	Auxin-like 1 protein
GLYMA02G13230	GLYMA02G13230.3	2	11487128	C	A	1840C>A	Gln614Lys	AT1G75310	Auxin-like 1 protein
GLYMA02G13230	GLYMA02G13230.3	2	11487132	C	T	1844C>T	Thr615Ile	AT1G75310	Auxin-like 1 protein
GLYMA02G13230	GLYMA02G13230.3	2	11487255	C	A	1967C>A	Ala656Asp	AT1G75310	Auxin-like 1 protein
GLYMA02G13230	GLYMA02G13230.3	2	11487830	G	A	2542G>A	Val848Ile	AT1G75310	Auxin-like 1 protein
GLYMA02G13230	GLYMA02G13230.3	2	11488113	A	C	2825A>C	Asn942Thr	AT1G75310	Auxin-like 1 protein
GLYMA02G13230	GLYMA02G13230.3	2	11488158	T	C	2870T>C	Phe957Ser	AT1G75310	Auxin-like 1 protein
GLYMA02G13230	GLYMA02G13230.3	2	11488242	T	C	2954T>C	Leu985Pro	AT1G75310	Auxin-like 1 protein
GLYMA02G13230	GLYMA02G13230.3	2	11489163	C	G	3670C>G	His1224Asp	AT1G75310	Auxin-like 1 protein
GLYMA02G13230	GLYMA02G13230.3	2	11489226	C	A	3733C>A	Leu1245Ile	AT1G75310	Auxin-like 1 protein
GLYMA02G16020	GLYMA02G16020.1	2	14452019	C	A	1073G>T	Trp358Leu	AT1G06800	Alpha/beta-Hydrolases superfamily protein
GLYMA02G16020	GLYMA02G16020.1	2	14452035	G	A	1057C>T	Pro353Ser	AT1G06800	Alpha/beta-Hydrolases superfamily protein
GLYMA02G16020	GLYMA02G16020.1	2	14452040	C	A	1052G>T	Trp351Leu	AT1G06800	Alpha/beta-Hydrolases superfamily protein
GLYMA02G16020	GLYMA02G16020.1	2	14452578	G	T	514C>A	His172Asn	AT1G06800	Alpha/beta-Hydrolases superfamily protein
GLYMA02G16020	GLYMA02G16020.1	2	14452602	C	T	490G>A	Asp164Asn	AT1G06800	Alpha/beta-Hydrolases superfamily protein
GLYMA02G16020	GLYMA02G16020.1	2	14458445	A	T	388T>A	Cys130Ser	AT1G06800	Alpha/beta-Hydrolases superfamily protein
GLYMA02G30736	GLYMA02G30736.1	2	32806982	A	G	16A>G	Thr6Ala	N/A	N/A
GLYMA02G41964	GLYMA02G41964.1	2	47035065	G	A	182G>A	Arg61Gln	AT5G65640	Beta HLH protein 93
GLYMA02G38611	GLYMA02G38611.1	2	43997929	T	A	99T>A	Asp33Glu	ATCG00160	Ribosomal protein S2
GLYMA02G30736	GLYMA02G30736.1	2	32807022	C	A	56C>A	Pro19Gln	N/A	N/A

GLYMA02G31971	GLYMA02G31971.1	2	35217889	T	C	121A>G	Lys41Glu	ATCG00470	ATP synthase epsilon chain
GLYMA02G31971	GLYMA02G31971.1	2	35217963	A	G	47T>C	Ile16Thr	ATCG00470	ATP synthase epsilon chain
GLYMA02G34180	GLYMA02G34180.2	2	38377432	G	A	173C>T	Thr58Ile	AT5G13420	Aldolase-type TIM barrel family protein
GLYMA02G34180	GLYMA02G34180.2	2	38377469	T	C	136A>G	Ile46Val	AT5G13420	Aldolase-type TIM barrel family protein
GLYMA02G34180	GLYMA02G34180.2	2	38377594	C	T	11G>A	Gly4Glu	AT5G13420	Aldolase-type TIM barrel family protein
GLYMA02G37190	GLYMA02G37190.2	2	42522633	G	A	157G>A	Glu53Lys	AT1G19260	TTF-type zinc finger protein with HAT dimerisation domain
GLYMA02G37190	GLYMA02G37190.2	2	42522691	C	T	215C>T	Thr72Ile	AT1G19260	TTF-type zinc finger protein with HAT dimerisation domain
GLYMA02G37190	GLYMA02G37190.2	2	42522700	C	T	224C>T	Thr75Met	AT1G19260	TTF-type zinc finger protein with HAT dimerisation domain
GLYMA02G37190	GLYMA02G37190.2	2	42522754	T	C	278T>C	Val93Ala	AT1G19260	TTF-type zinc finger protein with HAT dimerisation domain
GLYMA02G37190	GLYMA02G37190.2	2	42522762	G	A	286G>A	Val96Ile	AT1G19260	TTF-type zinc finger protein with HAT dimerisation domain
GLYMA02G30736	GLYMA02G30736.1	2	32807940	A	G	332A>G	Lys111Arg	N/A	N/A
GLYMA02G38611	GLYMA02G38611.1	2	43997952	T	C	122T>C	Val41Ala	ATCG00160	ribosomal protein S2
GLYMA02G40876	GLYMA02G40876.1	2	46090883	C	A	169C>A	Gln57Lys	AT2G01990	N/A
GLYMA02G41964	GLYMA02G41964.1	2	47035048	G	A	165G>A	Met55Ile	AT5G65640	Beta HLH protein 93
GLYMA02G30736	GLYMA02G30736.1	2	32807942	G	T	334G>T	Asp112Tyr	N/A	N/A
GLYMA02G41964	GLYMA02G41964.1	2	47035354	T	A	388T>A	Ser130Thr	AT5G65640	Beta HLH protein 93
GLYMA02G42410	GLYMA02G42410.1	2	47444326	A	G	880A>G	Asn294Asp	AT2G40116	Phosphoinositide-specific phospholipase C family protein
GLYMA03G00540	GLYMA03G00540.1	3	290319	G	C	589G>C	Val197Leu	AT4G00340	Receptor-like protein kinase 4
GLYMA03G00540	GLYMA03G00540.1	3	290434	A	C	704A>C	Tyr235Ser	AT4G00340	Receptor-like protein kinase 4
GLYMA03G00540	GLYMA03G00540.1	3	290578	G	A	848G>A	Arg283Lys	AT4G00340	Receptor-like protein kinase 4
GLYMA03G05281	GLYMA03G05281.1	3	5565886	A	T	128A>T	Gln43Leu	AT1G79630	Protein phosphatase

									2C family protein
GLYMA03G05281	GLYMA03G05281.1	3	5566105	C	A	347C>A	Ser116Tyr	AT1G79630	Protein phosphatase 2C family protein
GLYMA03G05281	GLYMA03G05281.1	3	5566121	T	G	363T>G	Phe121Leu	AT1G79630	Protein phosphatase 2C family protein
GLYMA03G05400	GLYMA03G05400.1	3	5646893	G	A	436G>A	Ala146Thr	AT3G14470	NB-ARC domain-containing disease resistance protein
GLYMA03G05400	GLYMA03G05400.1	3	5646979	G	T	522G>T	Glu174Asp	AT3G14470	NB-ARC domain-containing disease resistance protein
GLYMA03G05400	GLYMA03G05400.1	3	5647398	T	C	856T>C	Tyr286His	AT3G14470	NB-ARC domain-containing disease resistance protein
GLYMA03G05400	GLYMA03G05400.1	3	5647399	A	T	857A>T	Tyr286Phe	AT3G14470	NB-ARC domain-containing disease resistance protein
GLYMA03G05400	GLYMA03G05400.1	3	5647584	A	G	1042A>G	Thr348Ala	AT3G14470	NB-ARC domain-containing disease resistance protein
GLYMA03G06734	GLYMA03G06734.1	3	7018799	C	T	317C>T	Thr106Ile	AT5G10770	Eukaryotic aspartyl protease family protein
GLYMA03G06734	GLYMA03G06734.1	3	7018813	A	G	331A>G	Arg111Gly	AT5G10770	Eukaryotic aspartyl protease family protein
GLYMA03G36221	GLYMA03G36221.1	3	43237642	T	C	1745T>C	Ile582Thr	AT5G06440	N/A
GLYMA03G37870	GLYMA03G37870.1	3	44338697	C	T	113C>T	Thr38Ile	AT2G34830	WRKY DNA-binding protein 35
GLYMA03G37870	GLYMA03G37870.1	3	44338740	G	C	156G>C	Lys52Asn	AT2G34830	WRKY DNA-binding protein 35
GLYMA03G37870	GLYMA03G37870.1	3	44338757	C	T	173C>T	Ser58Leu	AT2G34830	WRKY DNA-binding protein 35
GLYMA03G37870	GLYMA03G37870.1	3	44338768	T	G	184T>G	Ser62Ala	AT2G34830	WRKY DNA-binding protein 35
GLYMA03G37870	GLYMA03G37870.1	3	44338791	A	T	207A>T	Lys69Asn	AT2G34830	WRKY DNA-binding protein 35
GLYMA03G37870	GLYMA03G37870.1	3	44339396	G	C	286G>C	Glu96Gln	AT2G34830	WRKY DNA-binding protein 35
GLYMA03G37870	GLYMA03G37870.1	3	44339409	C	T	299C>T	Thr100Met	AT2G34830	WRKY DNA-binding protein 35
GLYMA04G09780	GLYMA04G09780.2	4	8040286	A	G	2513T>C	Leu838Pro	AT4G24580	Rho GTPase activation protein (RhoGAP) with PH domain

GLYMA04G09780	GLYMA04G09780.2	4	8040307	G	T	2492C>A	Ser831Tyr	AT4G24580	Rho GTPase activation protein (RhoGAP) with PH domain
GLYMA04G09780	GLYMA04G09780.2	4	8043172	A	T	1573T>A	Tyr525Asn	AT4G24580	Rho GTPase activation protein (RhoGAP) with PH domain
GLYMA04G09780	GLYMA04G09780.3	4	8040286	A	G	2492T>C	Leu831Pro	AT4G24580	Rho GTPase activation protein (RhoGAP) with PH domain
GLYMA04G09780	GLYMA04G09780.3	4	8040307	G	T	2471C>A	Ser824Tyr	AT4G24580	Rho GTPase activation protein (RhoGAP) with PH domain
GLYMA04G09780	GLYMA04G09780.3	4	8043172	A	T	1573T>A	Tyr525Asn	AT4G24580	Rho GTPase activation protein (RhoGAP) with PH domain
GLYMA04G09780	GLYMA04G09780.4	4	8040286	A	G	2462T>C	Leu821Pro	AT4G24580	Rho GTPase activation protein (RhoGAP) with PH domain
GLYMA04G09780	GLYMA04G09780.4	4	8040307	G	T	2441C>A	Ser814Tyr	AT4G24580	Rho GTPase activation protein (RhoGAP) with PH domain
GLYMA04G09780	GLYMA04G09780.4	4	8043172	A	T	1522T>A	Tyr508Asn	AT4G24580	Rho GTPase activation protein (RhoGAP) with PH domain
GLYMA04G09780	GLYMA04G09780.5	4	8040286	A	G	2453T>C	Leu818Pro	AT4G24580	Rho GTPase activation protein (RhoGAP) with PH domain
GLYMA04G09780	GLYMA04G09780.5	4	8040307	G	T	2432C>A	Ser811Tyr	AT4G24580	Rho GTPase activation protein (RhoGAP) with PH domain
GLYMA04G09780	GLYMA04G09780.5	4	8043172	A	T	1573T>A	Tyr525Asn	AT4G24580	Rho GTPase activation protein (RhoGAP) with PH domain

GLYMA04G09780	GLYMA04G09780.6	4	8040286	A	G	2441T>C	Leu814Pro	AT4G24580	Rho GTPase activation protein (RhoGAP) with PH domain
GLYMA04G09780	GLYMA04G09780.6	4	8040307	G	T	2420C>A	Ser807Tyr	AT4G24580	Rho GTPase activation protein (RhoGAP) with PH domain
GLYMA04G09780	GLYMA04G09780.6	4	8043172	A	T	1522T>A	Tyr508Asn	AT4G24580	Rho GTPase activation protein (RhoGAP) with PH domain
GLYMA04G09780	GLYMA04G09780.7	4	8040286	A	G	2402T>C	Leu801Pro	AT4G24580	Rho GTPase activation protein (RhoGAP) with PH domain
GLYMA04G09780	GLYMA04G09780.7	4	8040307	G	T	2381C>A	Ser794Tyr	AT4G24580	Rho GTPase activation protein (RhoGAP) with PH domain
GLYMA04G09780	GLYMA04G09780.7	4	8043172	A	T	1522T>A	Tyr508Asn	AT4G24580	Rho GTPase activation protein (RhoGAP) with PH domain
GLYMA04G09780	GLYMA04G09780.8	4	8040286	A	G	1847T>C	Leu616Pro	AT4G24580	Rho GTPase activation protein (RhoGAP) with PH domain
GLYMA04G09780	GLYMA04G09780.8	4	8040307	G	T	1826C>A	Ser609Tyr	AT4G24580	Rho GTPase activation protein (RhoGAP) with PH domain
GLYMA04G09780	GLYMA04G09780.8	4	8043172	A	T	967T>A	Tyr323Asn	AT4G24580	Rho GTPase activation protein (RhoGAP) with PH domain
GLYMA04G33080	GLYMA04G33080.1	4	38522708	C	A	1225C>A	Leu409Ile	AT1G68710	ATPase E1-E2 type family protein / haloacid dehalogenase-like hydrolase family protein
GLYMA04G33080	GLYMA04G33080.1	4	38522964	G	T	1481G>T	Ser494Ile	AT1G68710	ATPase E1-E2 type family protein / haloacid dehalogenase-

									like hydrolase family protein
GLYMA04G14770	GLYMA04G14770.1	4	14966105	C	T	1895C>T	Ala632Val	AT1G31810	Formin Homology 14
GLYMA04G14770	GLYMA04G14770.1	4	14966207	A	C	1997A>C	His666Pro	AT1G31810	Formin Homology 14
GLYMA04G14770	GLYMA04G14770.2	4	14966105	C	T	1895C>T	Ala632Val	AT1G31810	Formin Homology 14
GLYMA04G14770	GLYMA04G14770.2	4	14966207	A	C	1997A>C	His666Pro	AT1G31810	Formin Homology 14
GLYMA04G12220	GLYMA04G12220.2	4	11196701	A	T	2151T>A	Phe717Leu	AT4G10120	Sucrose-phosphate synthase family protein
GLYMA04G12220	GLYMA04G12220.2	4	11196964	C	A	1888G>T	Asp630Tyr	AT4G10120	Sucrose-phosphate synthase family protein
GLYMA04G12220	GLYMA04G12220.2	4	11197161	G	C	1804C>G	His602Asp	AT4G10120	Sucrose-phosphate synthase family protein
GLYMA04G12220	GLYMA04G12220.2	4	11197226	T	C	1739A>G	Gln580Arg	AT4G10120	Sucrose-phosphate synthase family protein
GLYMA04G12220	GLYMA04G12220.2	4	11197690	C	T	1672G>A	Val558Ile	AT4G10120	Sucrose-phosphate synthase family protein
GLYMA04G12220	GLYMA04G12220.2	4	11197774	C	T	1588G>A	Ala530Thr	AT4G10120	Sucrose-phosphate synthase family protein
GLYMA04G12220	GLYMA04G12220.2	4	11197777	A	G	1585T>C	Tyr529His	AT4G10120	Sucrose-phosphate synthase family protein
GLYMA04G12220	GLYMA04G12220.2	4	11197795	A	C	1567T>G	Tyr523Asp	AT4G10120	Sucrose-phosphate synthase family protein
GLYMA04G12220	GLYMA04G12220.2	4	11197837	C	T	1525G>A	Gly509Ser	AT4G10120	Sucrose-phosphate synthase family protein
GLYMA04G12220	GLYMA04G12220.2	4	11197928	C	T	1434G>A	Met478Ile	AT4G10120	Sucrose-phosphate synthase family protein
GLYMA04G12220	GLYMA04G12220.2	4	11197968	A	G	1394T>C	Val465Ala	AT4G10120	Sucrose-phosphate synthase family protein
GLYMA04G12220	GLYMA04G12220.2	4	11197978	C	T	1384G>A	Val462Met	AT4G10120	Sucrose-phosphate synthase family protein
GLYMA04G12220	GLYMA04G12220.2	4	11198040	G	A	1322C>T	Ala441Val	AT4G10120	Sucrose-phosphate synthase family protein
GLYMA04G12220	GLYMA04G12220.2	4	11198101	C	T	1261G>A	Gly421Ser	AT4G10120	Sucrose-phosphate synthase family protein
GLYMA04G12220	GLYMA04G12220.2	4	11198154	A	G	1208T>C	Val403Ala	AT4G10120	Sucrose-phosphate synthase family protein
GLYMA04G12220	GLYMA04G12220.2	4	11200087	G	T	594C>A	Asp198Glu	AT4G10120	Sucrose-phosphate synthase family protein
GLYMA04G12220	GLYMA04G12220.2	4	11198326	G	A	1138C>T	Arg380Cys	AT4G10120	Sucrose-phosphate synthase family protein
GLYMA04G12220	GLYMA04G12220.2	4	11198668	C	T	953G>A	Gly318Glu	AT4G10120	Sucrose-phosphate synthase family protein

GLYMA04G12220	GLYMA04G12220.2	4	11198687	C	T	934G>A	Val312Ile	AT4G10120	Sucrose-phosphate synthase family protein
GLYMA04G12220	GLYMA04G12220.2	4	11200356	C	T	406G>A	Glu136Lys	AT4G10120	Sucrose-phosphate synthase family protein
GLYMA04G12220	GLYMA04G12220.2	4	11200385	G	A	377C>T	Thr126Ile	AT4G10120	Sucrose-phosphate synthase family protein
GLYMA04G12220	GLYMA04G12220.2	4	11198884	T	C	880A>G	Met294Val	AT4G10120	Sucrose-phosphate synthase family protein
GLYMA04G12220	GLYMA04G12220.2	4	11198890	T	C	874A>G	Thr292Ala	AT4G10120	Sucrose-phosphate synthase family protein
GLYMA04G12220	GLYMA04G12220.2	4	11200611	T	C	151A>G	Ser51Gly	AT4G10120	Sucrose-phosphate synthase family protein
GLYMA04G12220	GLYMA04G12220.2	4	11200076	G	A	605C>T	Ala202Val	AT4G10120	Sucrose-phosphate synthase family protein
GLYMA04G12220	GLYMA04G12220.2	4	11200788	T	C	55A>G	Lys19Glu	AT4G10120	Sucrose-phosphate synthase family protein
GLYMA04G12220	GLYMA04G12220.2	4	11200790	C	G	53G>C	Gly18Ala	AT4G10120	Sucrose-phosphate synthase family protein
GLYMA04G12220	GLYMA04G12220.2	4	11200095	C	T	586G>A	Gly196Ser	AT4G10120	Sucrose-phosphate synthase family protein
GLYMA04G12220	GLYMA04G12220.2	4	11200227	T	C	535A>G	Arg179Gly	AT4G10120	Sucrose-phosphate synthase family protein
GLYMA04G14770	GLYMA04G14770.1	4	14966251	G	A	2041G>A	Gly681Ser	AT1G31810	Formin Homology 14
GLYMA04G12220	GLYMA04G12220.2	4	11200256	T	A	506A>T	Glu169Val	AT4G10120	Sucrose-phosphate synthase family protein
GLYMA04G12220	GLYMA04G12220.2	4	11200533	C	T	229G>A	Gly77Ser	AT4G10120	Sucrose-phosphate synthase family protein
GLYMA04G14770	GLYMA04G14770.2	4	14966251	G	A	2041G>A	Gly681Ser	AT1G31810	Formin Homology 14
GLYMA04G12220	GLYMA04G12220.2	4	11200535	G	T	227C>A	Pro76His	AT4G10120	Sucrose-phosphate synthase family protein
GLYMA04G32381	GLYMA04G32381.1	4	37016724	T	G	245T>G	Ile82Arg	AT1G53035	N/A
GLYMA04G12220	GLYMA04G12220.2	4	11200763	A	G	80T>C	Val27Ala	AT4G10120	Sucrose-phosphate synthase family protein
GLYMA04G32381	GLYMA04G32381.1	4	37016829	G	T	305G>T	Trp102Leu	AT1G53035	N/A
GLYMA04G33080	GLYMA04G33080.1	4	38522393	A	G	910A>G	Ile304Val	AT1G68710	ATPase E1-E2 type family protein / haloacid dehalogenase-like hydrolase family protein
GLYMA04G32381	GLYMA04G32381.1	4	37016691	G	T	212G>T	Cys71Phe	AT1G53035	N/A
GLYMA04G32381	GLYMA04G32381.1	4	37016728	C	G	249C>G	Asp83Glu	AT1G53035	N/A

GLYMA05G18342	GLYMA05G18342.1	5	21842369	A	G	329A>G	Glu110Gly	ATMG00220	Apocytochrome b
GLYMA05G18342	GLYMA05G18342.1	5	21842401	A	G	361A>G	Thr121Ala	ATMG00220	Apocytochrome b
GLYMA05G18342	GLYMA05G18342.1	5	21842453	T	C	413T>C	Ile138Thr	ATMG00220	Apocytochrome b
GLYMA05G18342	GLYMA05G18342.1	5	21842603	A	T	563A>T	His188Leu	ATMG00220	Apocytochrome b
GLYMA05G18342	GLYMA05G18342.1	5	21842617	A	G	577A>G	Thr193Ala	ATMG00220	Apocytochrome b
GLYMA05G18342	GLYMA05G18342.1	5	21842656	T	C	616T>C	Tyr206His	ATMG00220	Apocytochrome b
GLYMA05G18342	GLYMA05G18342.1	5	21842878	A	G	838A>G	Asn280Asp	ATMG00220	Apocytochrome b
GLYMA05G18342	GLYMA05G18342.1	5	21842905	A	G	865A>G	Thr289Ala	ATMG00220	Apocytochrome b
GLYMA05G18342	GLYMA05G18342.1	5	21842974	T	C	895T>C	Cys299Arg	ATMG00220	Apocytochrome b
GLYMA05G18342	GLYMA05G18342.1	5	21843110	T	C	1031T>C	Val344Ala	ATMG00220	Apocytochrome b
GLYMA05G18342	GLYMA05G18342.1	5	21843125	T	C	1046T>C	Leu349Pro	ATMG00220	Apocytochrome b
GLYMA05G18342	GLYMA05G18342.1	5	21843172	A	G	1093A>G	Asn365Asp	ATMG00220	Apocytochrome b
GLYMA05G33610	GLYMA05G33610.1	5	38230518	T	C	449A>G	Gln150Arg	AT1G73480	Alpha/beta-Hydrolases superfamily protein
GLYMA05G33610	GLYMA05G33610.1	5	38230539	T	C	428A>G	Lys143Arg	AT1G73480	Alpha/beta-Hydrolases superfamily protein
GLYMA05G33610	GLYMA05G33610.2	5	38230518	T	C	575A>G	Gln192Arg	AT1G73480	Alpha/beta-Hydrolases superfamily protein
GLYMA05G33610	GLYMA05G33610.2	5	38230539	T	C	554A>G	Lys185Arg	AT1G73480	Alpha/beta-Hydrolases superfamily protein
GLYMA05G33610	GLYMA05G33610.3	5	38230518	T	C	575A>G	Gln192Arg	AT1G73480	Alpha/beta-Hydrolases superfamily protein
GLYMA05G33610	GLYMA05G33610.3	5	38230539	T	C	554A>G	Lys185Arg	AT1G73480	Alpha/beta-Hydrolases superfamily protein
GLYMA05G33610	GLYMA05G33610.4	5	38230518	T	C	449A>G	Gln150Arg	AT1G73480	Alpha/beta-Hydrolases superfamily protein
GLYMA05G33610	GLYMA05G33610.4	5	38230539	T	C	428A>G	Lys143Arg	AT1G73480	Alpha/beta-Hydrolases superfamily protein
GLYMA05G33610	GLYMA05G33610.5	5	38230518	T	C	449A>G	Gln150Arg	AT1G73480	Alpha/beta-Hydrolases superfamily protein
GLYMA05G33610	GLYMA05G33610.5	5	38230539	T	C	428A>G	Lys143Arg	AT1G73480	Alpha/beta-Hydrolases superfamily protein
GLYMA05G33610	GLYMA05G33610.6	5	38230518	T	C	449A>G	Gln150Arg	AT1G73480	Alpha/beta-Hydrolases superfamily protein
GLYMA05G33610	GLYMA05G33610.6	5	38230539	T	C	428A>G	Lys143Arg	AT1G73480	Alpha/beta-Hydrolases superfamily protein
GLYMA06G10320	GLYMA06G10320.1	6	7807413	T	A	3566A>T	Glu1189Val	AT1G58250	Golgi-body localisation protein

									domain ;RNA pol II promoter Fmp27 protein domain
GLYMA06G10320	GLYMA06G10320.1	6	7818931	C	T	394G>A	Asp132Asn	AT1G58250	Golgi-body localisation protein domain ;RNA pol II promoter Fmp27 protein domain
GLYMA06G10320	GLYMA06G10320.1	6	7818960	A	G	365T>C	Met122Thr	AT1G58250	Golgi-body localisation protein domain ;RNA pol II promoter Fmp27 protein domain
GLYMA06G10320	GLYMA06G10320.1	6	7819065	A	G	260T>C	Val87Ala	AT1G58250	Golgi-body localisation protein domain ;RNA pol II promoter Fmp27 protein domain
GLYMA06G10320	GLYMA06G10320.1	6	7819111	C	T	214G>A	Gly72Arg	AT1G58250	Golgi-body localisation protein domain ;RNA pol II promoter Fmp27 protein domain
GLYMA06G10320	GLYMA06G10320.2	6	7807413	T	A	3878A>T	Glu1293Val	AT1G58250	Golgi-body localisation protein domain ;RNA pol II promoter Fmp27 protein domain
GLYMA06G10320	GLYMA06G10320.2	6	7818931	C	T	706G>A	Asp236Asn	AT1G58250	Golgi-body localisation protein domain ;RNA pol II promoter Fmp27 protein domain
GLYMA06G10320	GLYMA06G10320.2	6	7818960	A	G	677T>C	Met226Thr	AT1G58250	Golgi-body localisation protein domain ;RNA pol II promoter Fmp27 protein domain
GLYMA06G10320	GLYMA06G10320.2	6	7819065	A	G	572T>C	Val191Ala	AT1G58250	Golgi-body localisation protein domain ;RNA pol II promoter Fmp27 protein domain
GLYMA06G10320	GLYMA06G10320.2	6	7819111	C	T	526G>A	Gly176Arg	AT1G58250	Golgi-body

									localisation protein domain ;RNA pol II promoter Fmp27 protein domain
GLYMA06G42661	GLYMA06G42661.1	6	45947027	G	C	1157C>G	Pro386Arg	AT5G43060	Granulin repeat cysteine protease family protein
GLYMA06G20100	GLYMA06G20100.1	6	16470075	T	A	847A>T	Ile283Phe	AT1G21720	Proteasome beta subunit C1
GLYMA06G20100	GLYMA06G20100.1	6	16470170	C	T	752G>A	Ser251Asn	AT1G21720	Proteasome beta subunit C1
GLYMA06G42661	GLYMA06G42661.1	6	45953624	A	G	95T>C	Ile32Thr	AT5G43060	Granulin repeat cysteine protease family protein
GLYMA06G38901	GLYMA06G38901.1	6	41867119	C	T	509C>T	Ser170Leu	AT5G09880	Splicing factor, CC1-like
GLYMA06G38901	GLYMA06G38901.1	6	41867169	G	A	559G>A	Val187Ile	AT5G09880	Splicing factor, CC1-like
GLYMA06G41880	GLYMA06G41880.1	6	45152943	T	C	656T>C	Val219Ala	AT5G17680	Disease resistance protein (TIR-NBS-LRR class), putative
GLYMA06G41880	GLYMA06G41880.1	6	45152957	G	A	670G>A	Glu224Lys	AT5G17680	Disease resistance protein (TIR-NBS-LRR class), putative
GLYMA06G41880	GLYMA06G41880.1	6	45153007	C	A	720C>A	His240Gln	AT5G17680	Disease resistance protein (TIR-NBS-LRR class), putative
GLYMA06G41880	GLYMA06G41880.1	6	45153142	C	A	855C>A	Ser285Arg	AT5G17680	Disease resistance protein (TIR-NBS-LRR class), putative
GLYMA06G41880	GLYMA06G41880.1	6	45153228	G	T	941G>T	Gly314Val	AT5G17680	disease resistance protein (TIR-NBS-LRR class), putative
GLYMA06G42661	GLYMA06G42661.1	6	45953627	T	G	92A>C	Lys31Thr	AT5G43060	Granulin repeat cysteine protease family protein
GLYMA06G42661	GLYMA06G42661.1	6	45948528	T	G	619A>C	Ile207Leu	AT5G43060	Granulin repeat cysteine protease family protein
GLYMA06G20100	GLYMA06G20100.1	6	16470054	C	T	868G>A	Ala290Thr	AT1G21720	proteasome beta subunit C1
GLYMA06G38901	GLYMA06G38901.1	6	41867089	G	T	479G>T	Cys160Phe	AT5G09880	Splicing factor, CC1-like

GLYMA06G43200	GLYMA06G43200.2	6	46492903	A	G	209T>C	Leu70Ser	AT4G38840	SAUR-like auxin-responsive protein family
GLYMA06G47320	GLYMA06G47320.2	6	49813468	C	T	896C>T	Ala299Val	AT5G04890	HSP20-like chaperones superfamily protein
GLYMA06G47320	GLYMA06G47320.2	6	49813618	T	C	1046T>C	Ile349Thr	AT5G04890	HSP20-like chaperones superfamily protein
GLYMA06G47320	GLYMA06G47320.2	6	49813642	C	T	1070C>T	Thr357Ile	AT5G04890	HSP20-like chaperones superfamily protein
GLYMA07G06866	GLYMA07G06866.1	7	5532808	C	G	5818G>C	Val1940Leu	AT4G27220	NB-ARC domain-containing disease resistance protein
GLYMA07G06866	GLYMA07G06866.1	7	5533251	C	T	5375G>A	Cys1792Tyr	AT4G27220	NB-ARC domain-containing disease resistance protein
GLYMA07G06866	GLYMA07G06866.1	7	5533420	G	T	5206C>A	Gln1736Lys	AT4G27220	NB-ARC domain-containing disease resistance protein
GLYMA07G06866	GLYMA07G06866.1	7	5533739	C	G	5159G>C	Cys1720Ser	AT4G27220	NB-ARC domain-containing disease resistance protein
GLYMA07G06866	GLYMA07G06866.1	7	5533786	G	C	5112C>G	Ser1704Arg	AT4G27220	NB-ARC domain-containing disease resistance protein
GLYMA07G06866	GLYMA07G06866.1	7	5533947	C	T	4951G>A	Glu1651Lys	AT4G27220	NB-ARC domain-containing disease resistance protein
GLYMA07G06866	GLYMA07G06866.1	7	5539466	G	C	3045C>G	Ser1015Arg	AT4G27220	NB-ARC domain-containing disease resistance protein
GLYMA07G07015	GLYMA07G07015.1	7	5653900	C	A	484C>A	Gln162Lys	AT4G27220	NB-ARC domain-containing disease resistance protein
GLYMA07G07015	GLYMA07G07015.1	7	5658404	T	G	4075T>G	Leu1359Val	AT4G27220	NB-ARC domain-containing disease resistance protein
GLYMA07G07015	GLYMA07G07015.1	7	5658406	G	T	4077G>T	Leu1359Phe	AT4G27220	NB-ARC domain-containing disease resistance protein
GLYMA07G07015	GLYMA07G07015.1	7	5658443	T	A	4114T>A	Trp1372Arg	AT4G27220	NB-ARC domain-containing disease

									resistance protein
GLYMA07G07015	GLYMA07G07015.1	7	5658453	C	T	4124C>T	Thr1375Ile	AT4G27220	NB-ARC domain-containing disease resistance protein
GLYMA07G07015	GLYMA07G07015.1	7	5662433	A	G	4957A>G	Lys1653Glu	AT4G27220	NB-ARC domain-containing disease resistance protein
GLYMA07G07015	GLYMA07G07015.1	7	5662436	A	G	4960A>G	Lys1654Glu	AT4G27220	NB-ARC domain-containing disease resistance protein
GLYMA07G07015	GLYMA07G07015.1	7	5662449	A	T	4973A>T	Glu1658Val	AT4G27220	NB-ARC domain-containing disease resistance protein
GLYMA07G07015	GLYMA07G07015.1	7	5666667	C	T	6580C>T	His2194Tyr	AT4G27220	NB-ARC domain-containing disease resistance protein
GLYMA07G07015	GLYMA07G07015.1	7	5666737	A	T	6650A>T	Lys2217Met	AT4G27220	NB-ARC domain-containing disease resistance protein
GLYMA07G07015	GLYMA07G07015.1	7	5666752	C	G	6665C>G	Ala2222Gly	AT4G27220	NB-ARC domain-containing disease resistance protein
GLYMA07G07015	GLYMA07G07015.1	7	5666836	C	A	6749C>A	Thr2250Lys	AT4G27220	NB-ARC domain-containing disease resistance protein
GLYMA07G07015	GLYMA07G07015.1	7	5666842	C	T	6755C>T	Thr2252Ile	AT4G27220	NB-ARC domain-containing disease resistance protein
GLYMA07G07075	GLYMA07G07075.1	7	5697894	C	T	928C>T	Pro310Ser	AT4G27220	NB-ARC domain-containing disease resistance protein
GLYMA07G07075	GLYMA07G07075.1	7	5698025	T	A	1059T>A	Asp353Glu	AT4G27220	NB-ARC domain-containing disease resistance protein
GLYMA07G07075	GLYMA07G07075.1	7	5698070	G	C	1104G>C	Gln368His	AT4G27220	NB-ARC domain-containing disease resistance protein
GLYMA07G07075	GLYMA07G07075.1	7	5698752	T	A	1786T>A	Ser596Thr	AT4G27220	NB-ARC domain-containing disease resistance protein
GLYMA07G07075	GLYMA07G07075.1	7	5698808	G	C	1842G>C	Glu614Asp	AT4G27220	NB-ARC domain-containing disease resistance protein

GLYMA07G07075	GLYMA07G07075.1	7	5699242	A	T	2276A>T	Lys759Met	AT4G27220	NB-ARC domain-containing disease resistance protein
GLYMA07G07075	GLYMA07G07075.1	7	5699289	G	A	2323G>A	Asp775Asn	AT4G27220	NB-ARC domain-containing disease resistance protein
GLYMA07G07075	GLYMA07G07075.1	7	5699332	G	C	2366G>C	Arg789Thr	AT4G27220	NB-ARC domain-containing disease resistance protein
GLYMA07G07075	GLYMA07G07075.1	7	5699650	C	T	2684C>T	Ser895Phe	AT4G27220	NB-ARC domain-containing disease resistance protein
GLYMA07G07075	GLYMA07G07075.1	7	5699671	T	C	2705T>C	Val902Ala	AT4G27220	NB-ARC domain-containing disease resistance protein
GLYMA07G07075	GLYMA07G07075.1	7	5699767	A	T	2801A>T	Lys934Met	AT4G27220	NB-ARC domain-containing disease resistance protein
GLYMA07G07075	GLYMA07G07075.1	7	5699781	C	G	2815C>G	Gln939Glu	AT4G27220	NB-ARC domain-containing disease resistance protein
GLYMA07G07075	GLYMA07G07075.1	7	5701585	G	A	3958G>A	Glu1320Lys	AT4G27220	NB-ARC domain-containing disease resistance protein
GLYMA07G07075	GLYMA07G07075.2	7	5697894	C	T	928C>T	Pro310Ser	AT4G27220	NB-ARC domain-containing disease resistance protein
GLYMA07G07075	GLYMA07G07075.2	7	5698025	T	A	1059T>A	Asp353Glu	AT4G27220	NB-ARC domain-containing disease resistance protein
GLYMA07G07075	GLYMA07G07075.2	7	5698070	G	C	1104G>C	Gln368His	AT4G27220	NB-ARC domain-containing disease resistance protein
GLYMA07G07075	GLYMA07G07075.2	7	5698752	T	A	1786T>A	Ser596Thr	AT4G27220	NB-ARC domain-containing disease resistance protein
GLYMA07G07075	GLYMA07G07075.2	7	5698808	G	C	1842G>C	Glu614Asp	AT4G27220	NB-ARC domain-containing disease resistance protein
GLYMA07G07075	GLYMA07G07075.2	7	5699242	A	T	2276A>T	Lys759Met	AT4G27220	NB-ARC domain-containing disease resistance protein
GLYMA07G07075	GLYMA07G07075.2	7	5699289	G	A	2323G>A	Asp775Asn	AT4G27220	NB-ARC domain-containing disease

									resistance protein
GLYMA07G07075	GLYMA07G07075.2	7	5699332	G	C	2366G>C	Arg789Thr	AT4G27220	NB-ARC domain-containing disease resistance protein
GLYMA07G07075	GLYMA07G07075.2	7	5699650	C	T	2684C>T	Ser895Phe	AT4G27220	NB-ARC domain-containing disease resistance protein
GLYMA07G07075	GLYMA07G07075.2	7	5699671	T	C	2705T>C	Val902Ala	AT4G27220	NB-ARC domain-containing disease resistance protein
GLYMA07G07075	GLYMA07G07075.2	7	5699767	A	T	2801A>T	Lys934Met	AT4G27220	NB-ARC domain-containing disease resistance protein
GLYMA07G07075	GLYMA07G07075.2	7	5699781	C	G	2815C>G	Gln939Glu	AT4G27220	NB-ARC domain-containing disease resistance protein
GLYMA07G07075	GLYMA07G07075.2	7	5701585	G	A	3958G>A	Glu1320Lys	AT4G27220	NB-ARC domain-containing disease resistance protein
GLYMA07G07100	GLYMA07G07100.2	7	5723690	T	G	13T>G	Leu5Val	AT4G27190	NB-ARC domain-containing disease resistance protein
GLYMA07G07100	GLYMA07G07100.2	7	5723724	C	T	47C>T	Ala16Val	AT4G27190	NB-ARC domain-containing disease resistance protein
GLYMA07G07100	GLYMA07G07100.2	7	5725793	G	A	2116G>A	Val706Met	AT4G27190	NB-ARC domain-containing disease resistance protein
GLYMA07G07100	GLYMA07G07100.2	7	5725896	T	A	2219T>A	Val740Asp	AT4G27190	NB-ARC domain-containing disease resistance protein
GLYMA07G07100	GLYMA07G07100.2	7	5727067	C	A	3192C>A	Asn1064Lys	AT4G27190	NB-ARC domain-containing disease resistance protein
GLYMA07G07100	GLYMA07G07100.2	7	5727651	A	T	3776A>T	Lys1259Met	AT4G27190	NB-ARC domain-containing disease resistance protein
GLYMA07G07100	GLYMA07G07100.2	7	5731896	C	G	4782C>G	Asn1594Lys	AT4G27190	NB-ARC domain-containing disease resistance protein
GLYMA07G07100	GLYMA07G07100.2	7	5731918	G	A	4804G>A	Gly1602Arg	AT4G27190	NB-ARC domain-containing disease resistance protein

GLYMA07G07100	GLYMA07G07100.2	7	5731968	G	C	4854G>C	Leu1618Phe	AT4G27190	NB-ARC domain-containing disease resistance protein
GLYMA07G07100	GLYMA07G07100.2	7	5731981	G	C	4867G>C	Glu1623Gln	AT4G27190	NB-ARC domain-containing disease resistance protein
GLYMA07G07100	GLYMA07G07100.2	7	5732062	T	A	4948T>A	Phe1650Ile	AT4G27190	NB-ARC domain-containing disease resistance protein
GLYMA07G07100	GLYMA07G07100.2	7	5733159	A	G	5417A>G	Tyr1806Cys	AT4G27190	NB-ARC domain-containing disease resistance protein
GLYMA07G07100	GLYMA07G07100.4	7	5723690	T	G	13T>G	Leu5Val	AT4G27190	NB-ARC domain-containing disease resistance protein
GLYMA07G07100	GLYMA07G07100.4	7	5723724	C	T	47C>T	Ala16Val	AT4G27190	NB-ARC domain-containing disease resistance protein
GLYMA07G07100	GLYMA07G07100.4	7	5725793	G	A	2116G>A	Val706Met	AT4G27190	NB-ARC domain-containing disease resistance protein
GLYMA07G07100	GLYMA07G07100.4	7	5725896	T	A	2219T>A	Val740Asp	AT4G27190	NB-ARC domain-containing disease resistance protein
GLYMA07G07100	GLYMA07G07100.4	7	5727067	C	A	3192C>A	Asn1064Lys	AT4G27190	NB-ARC domain-containing disease resistance protein
GLYMA07G07100	GLYMA07G07100.4	7	5727651	A	T	3776A>T	Lys1259Met	AT4G27190	NB-ARC domain-containing disease resistance protein
GLYMA07G07100	GLYMA07G07100.4	7	5731896	C	G	4782C>G	Asn1594Lys	AT4G27190	NB-ARC domain-containing disease resistance protein
GLYMA07G07100	GLYMA07G07100.4	7	5731918	G	A	4804G>A	Gly1602Arg	AT4G27190	NB-ARC domain-containing disease resistance protein
GLYMA07G07100	GLYMA07G07100.4	7	5731968	G	C	4854G>C	Leu1618Phe	AT4G27190	NB-ARC domain-containing disease resistance protein
GLYMA07G07100	GLYMA07G07100.4	7	5731981	G	C	4867G>C	Glu1623Gln	AT4G27190	NB-ARC domain-containing disease resistance protein
GLYMA07G07100	GLYMA07G07100.4	7	5732062	T	A	4948T>A	Phe1650Ile	AT4G27190	NB-ARC domain-containing disease

									resistance protein
GLYMA07G07100	GLYMA07G07100.4	7	5733159	A	G	5417A>G	Tyr1806Cys	AT4G27190	NB-ARC domain-containing disease resistance protein
GLYMA07G07100	GLYMA07G07100.5	7	5723690	T	G	13T>G	Leu5Val	AT4G27190	NB-ARC domain-containing disease resistance protein
GLYMA07G07100	GLYMA07G07100.5	7	5723724	C	T	47C>T	Ala16Val	AT4G27190	NB-ARC domain-containing disease resistance protein
GLYMA07G07100	GLYMA07G07100.5	7	5725793	G	A	2116G>A	Val706Met	AT4G27190	NB-ARC domain-containing disease resistance protein
GLYMA07G07100	GLYMA07G07100.5	7	5725896	T	A	2219T>A	Val740Asp	AT4G27190	NB-ARC domain-containing disease resistance protein
GLYMA07G07100	GLYMA07G07100.5	7	5727067	C	A	3192C>A	Asn1064Lys	AT4G27190	NB-ARC domain-containing disease resistance protein
GLYMA07G07100	GLYMA07G07100.5	7	5727651	A	T	3776A>T	Lys1259Met	AT4G27190	NB-ARC domain-containing disease resistance protein
GLYMA07G07100	GLYMA07G07100.5	7	5731896	C	G	4782C>G	Asn1594Lys	AT4G27190	NB-ARC domain-containing disease resistance protein
GLYMA07G07100	GLYMA07G07100.5	7	5731918	G	A	4804G>A	Gly1602Arg	AT4G27190	NB-ARC domain-containing disease resistance protein
GLYMA07G07100	GLYMA07G07100.5	7	5731968	G	C	4854G>C	Leu1618Phe	AT4G27190	NB-ARC domain-containing disease resistance protein
GLYMA07G07100	GLYMA07G07100.5	7	5731981	G	C	4867G>C	Glu1623Gln	AT4G27190	NB-ARC domain-containing disease resistance protein
GLYMA07G07100	GLYMA07G07100.5	7	5732062	T	A	4948T>A	Phe1650Ile	AT4G27190	NB-ARC domain-containing disease resistance protein
GLYMA07G07100	GLYMA07G07100.5	7	5733159	A	G	5417A>G	Tyr1806Cys	AT4G27190	NB-ARC domain-containing disease resistance protein
GLYMA07G07110	GLYMA07G07110.1	7	5765839	A	G	692A>G	Lys231Arg	AT4G27220	NB-ARC domain-containing disease resistance protein

GLYMA07G07110	GLYMA07G07110.1	7	5773674	G	A	4900G>A	Asp1634Asn	AT4G27220	NB-ARC domain-containing disease resistance protein
GLYMA07G07110	GLYMA07G07110.1	7	5773740	A	G	4966A>G	Lys1656Glu	AT4G27220	NB-ARC domain-containing disease resistance protein
GLYMA07G07110	GLYMA07G07110.1	7	5774851	G	A	5648G>A	Gly1883Asp	AT4G27220	NB-ARC domain-containing disease resistance protein
GLYMA07G07110	GLYMA07G07110.1	7	5774857	C	A	5654C>A	Ser1885Tyr	AT4G27220	NB-ARC domain-containing disease resistance protein
GLYMA07G07110	GLYMA07G07110.1	7	5774868	G	T	5665G>T	Asp1889Tyr	AT4G27220	NB-ARC domain-containing disease resistance protein
GLYMA07G07110	GLYMA07G07110.1	7	5774881	C	G	5678C>G	Ser1893Cys	AT4G27220	NB-ARC domain-containing disease resistance protein
GLYMA07G07110	GLYMA07G07110.1	7	5774928	A	G	5725A>G	Asn1909Asp	AT4G27220	NB-ARC domain-containing disease resistance protein
GLYMA07G07110	GLYMA07G07110.1	7	5774948	G	C	5745G>C	Leu1915Phe	AT4G27220	NB-ARC domain-containing disease resistance protein
GLYMA07G07110	GLYMA07G07110.1	7	5778777	G	A	7276G>A	Ala2426Thr	AT4G27220	NB-ARC domain-containing disease resistance protein
GLYMA07G07110	GLYMA07G07110.1	7	5778807	G	C	7306G>C	Glu2436Gln	AT4G27220	NB-ARC domain-containing disease resistance protein
GLYMA07G07150	GLYMA07G07150.1	7	5847784	T	A	823T>A	Leu275Ile	AT4G27190	NB-ARC domain-containing disease resistance protein
GLYMA07G07150	GLYMA07G07150.1	7	5851864	G	A	4405G>A	Asp1469Asn	AT4G27190	NB-ARC domain-containing disease resistance protein
GLYMA07G07150	GLYMA07G07150.1	7	5851883	A	C	4424A>C	Glu1475Ala	AT4G27190	NB-ARC domain-containing disease resistance protein
GLYMA07G07150	GLYMA07G07150.1	7	5851904	A	G	4445A>G	Lys1482Arg	AT4G27190	NB-ARC domain-containing disease resistance protein
GLYMA07G07150	GLYMA07G07150.1	7	5852077	G	T	4618G>T	Ala1540Ser	AT4G27190	NB-ARC domain-containing disease

									resistance protein
GLYMA07G07150	GLYMA07G07150.1	7	5852101	G	T	4642G>T	Ala1548Ser	AT4G27190	NB-ARC domain-containing disease resistance protein
GLYMA07G07150	GLYMA07G07150.2	7	5847784	T	A	823T>A	Leu275Ile	AT4G27190	NB-ARC domain-containing disease resistance protein
GLYMA07G07150	GLYMA07G07150.2	7	5851864	G	A	4405G>A	Asp1469Asn	AT4G27190	NB-ARC domain-containing disease resistance protein
GLYMA07G07150	GLYMA07G07150.2	7	5851883	A	C	4424A>C	Glu1475Ala	AT4G27190	NB-ARC domain-containing disease resistance protein
GLYMA07G07150	GLYMA07G07150.2	7	5851904	A	G	4445A>G	Lys1482Arg	AT4G27190	NB-ARC domain-containing disease resistance protein
GLYMA07G07150	GLYMA07G07150.2	7	5852077	G	T	4618G>T	Ala1540Ser	AT4G27190	NB-ARC domain-containing disease resistance protein
GLYMA07G07150	GLYMA07G07150.2	7	5852101	G	T	4642G>T	Ala1548Ser	AT4G27190	NB-ARC domain-containing disease resistance protein
GLYMA07G07150	GLYMA07G07150.4	7	5847784	T	A	823T>A	Leu275Ile	AT4G27190	NB-ARC domain-containing disease resistance protein
GLYMA07G07150	GLYMA07G07150.4	7	5851864	G	A	4405G>A	Asp1469Asn	AT4G27190	NB-ARC domain-containing disease resistance protein
GLYMA07G07150	GLYMA07G07150.4	7	5851883	A	C	4424A>C	Glu1475Ala	AT4G27190	NB-ARC domain-containing disease resistance protein
GLYMA07G07150	GLYMA07G07150.4	7	5851904	A	G	4445A>G	Lys1482Arg	AT4G27190	NB-ARC domain-containing disease resistance protein
GLYMA07G07150	GLYMA07G07150.4	7	5852077	G	T	4618G>T	Ala1540Ser	AT4G27190	NB-ARC domain-containing disease resistance protein
GLYMA07G07150	GLYMA07G07150.4	7	5852101	G	T	4642G>T	Ala1548Ser	AT4G27190	NB-ARC domain-containing disease resistance protein
GLYMA07G07150	GLYMA07G07150.5	7	5847784	T	A	823T>A	Leu275Ile	AT4G27190	NB-ARC domain-containing disease resistance protein

GLYMA07G07150	GLYMA07G07150.5	7	5851864	G	A	4405G>A	Asp1469Asn	AT4G27190	NB-ARC domain-containing disease resistance protein
GLYMA07G07150	GLYMA07G07150.5	7	5851883	A	C	4424A>C	Glu1475Ala	AT4G27190	NB-ARC domain-containing disease resistance protein
GLYMA07G07150	GLYMA07G07150.5	7	5851904	A	G	4445A>G	Lys1482Arg	AT4G27190	NB-ARC domain-containing disease resistance protein
GLYMA07G07150	GLYMA07G07150.5	7	5852077	G	T	4618G>T	Ala1540Ser	AT4G27190	NB-ARC domain-containing disease resistance protein
GLYMA07G07150	GLYMA07G07150.5	7	5852101	G	T	4642G>T	Ala1548Ser	AT4G27190	NB-ARC domain-containing disease resistance protein
GLYMA07G07320	GLYMA07G07320.1	7	6019270	A	T	195T>A	His65Gln	AT2G22590	UDP-Glycosyltransferase superfamily protein
GLYMA07G11031	GLYMA07G11031.1	7	9251843	A	T	107A>T	Lys36Met	N/A	N/A
GLYMA07G11031	GLYMA07G11031.1	7	9251853	T	G	117T>G	Phe39Leu	N/A	N/A
GLYMA07G11031	GLYMA07G11031.1	7	9251863	T	C	127T>C	Ser43Pro	N/A	N/A
GLYMA07G11031	GLYMA07G11031.1	7	9251885	A	G	149A>G	Lys50Arg	N/A	N/A
GLYMA07G11031	GLYMA07G11031.1	7	9251894	T	C	158T>C	Leu53Pro	N/A	N/A
GLYMA07G11031	GLYMA07G11031.1	7	9251905	C	T	169C>T	Pro57Ser	N/A	N/A
GLYMA07G11031	GLYMA07G11031.2	7	9251843	A	T	107A>T	Lys36Met	N/A	N/A
GLYMA07G11031	GLYMA07G11031.2	7	9251853	T	G	117T>G	Phe39Leu	N/A	N/A
GLYMA07G11031	GLYMA07G11031.2	7	9251863	T	C	127T>C	Ser43Pro	N/A	N/A
GLYMA07G11031	GLYMA07G11031.2	7	9251885	A	G	149A>G	Lys50Arg	N/A	N/A
GLYMA07G11031	GLYMA07G11031.2	7	9251894	T	C	158T>C	Leu53Pro	N/A	N/A
GLYMA07G11031	GLYMA07G11031.2	7	9251905	C	T	169C>T	Pro57Ser	N/A	N/A
GLYMA07G17370	GLYMA07G17370.2	7	17078731	T	C	1592A>G	Asp531Gly	AT1G74170	receptor like protein 13
GLYMA07G17370	GLYMA07G17370.2	7	17081358	T	C	967A>G	Lys323Glu	AT1G74170	receptor like protein 13
GLYMA07G17370	GLYMA07G17370.2	7	17081486	G	A	926C>T	Thr309Ile	AT1G74170	receptor like protein 13
GLYMA07G17370	GLYMA07G17370.2	7	17082443	T	C	403A>G	Ile135Val	AT1G74170	receptor like protein 13

GLYMA07G17370	GLYMA07G17370.2	7	17082446	C	A	400G>T	Ala134Ser	AT1G74170	receptor like protein 13
GLYMA07G17370	GLYMA07G17370.2	7	17082845	G	C	114C>G	Phe38Leu	AT1G74170	receptor like protein 13
GLYMA07G17370	GLYMA07G17370.2	7	17082886	G	A	73C>T	Arg25Cys	AT1G74170	receptor like protein 13
GLYMA07G17370	GLYMA07G17370.2	7	17082952	T	C	7A>G	Met3Val	AT1G74170	receptor like protein 13
GLYMA07G18010	GLYMA07G18010.1	7	17771284	A	G	1265A>G	Asn422Ser	AT3G18290	zinc finger protein-related
GLYMA07G18010	GLYMA07G18010.1	7	17771317	A	G	1298A>G	Tyr433Cys	AT3G18290	zinc finger protein-related
GLYMA07G18010	GLYMA07G18010.1	7	17771511	C	A	1371C>A	Asp457Glu	AT3G18290	zinc finger protein-related
GLYMA07G37265	GLYMA07G37265.1	7	42390659	T	C	260T>C	Leu87Pro	N/A	N/A
GLYMA07G37265	GLYMA07G37265.1	7	42390712	T	G	313T>G	Ser105Ala	N/A	N/A
GLYMA07G37265	GLYMA07G37265.1	7	42390731	C	A	332C>A	Ser111Tyr	N/A	N/A
GLYMA08G14790	GLYMA08G14790.3	8	10776565	C	G	434C>G	Ser145Cys	AT5G10060	ENTH/VHS family protein
GLYMA08G14790	GLYMA08G14790.4	8	10776565	C	G	434C>G	Ser145Cys	AT5G10060	ENTH/VHS family protein
GLYMA08G21140	GLYMA08G21140.2	8	16039317	C	T	1982G>A	Arg661Gln	AT4G29990	Leucine-rich repeat transmembrane protein kinase protein
GLYMA08G21140	GLYMA08G21140.2	8	16039487	C	A	1812G>T	Met604Ile	AT4G29990	Leucine-rich repeat transmembrane protein kinase protein
GLYMA08G21140	GLYMA08G21140.2	8	16039798	G	A	1736C>T	Ser579Leu	AT4G29990	Leucine-rich repeat transmembrane protein kinase protein
GLYMA08G21140	GLYMA08G21140.2	8	16039816	A	G	1718T>C	Val573Ala	AT4G29990	Leucine-rich repeat transmembrane protein kinase protein
GLYMA08G21140	GLYMA08G21140.2	8	16039825	A	G	1709T>C	Val570Ala	AT4G29990	Leucine-rich repeat transmembrane protein kinase protein
GLYMA08G21140	GLYMA08G21140.2	8	16039862	A	G	1672T>C	Cys558Arg	AT4G29990	Leucine-rich repeat transmembrane protein kinase protein
GLYMA08G21140	GLYMA08G21140.2	8	16042447	G	C	1342C>G	Leu448Val	AT4G29990	Leucine-rich repeat transmembrane protein kinase protein

GLYMA08G21140	GLYMA08G21140.2	8	16042675	T	C	1237A>G	Ile413Val	AT4G29990	Leucine-rich repeat transmembrane protein kinase protein
GLYMA08G21140	GLYMA08G21140.2	8	16042681	G	T	1231C>A	Gln411Lys	AT4G29990	Leucine-rich repeat transmembrane protein kinase protein
GLYMA08G21140	GLYMA08G21140.2	8	16042693	A	T	1219T>A	Phe407Ile	AT4G29990	Leucine-rich repeat transmembrane protein kinase protein
GLYMA08G21140	GLYMA08G21140.2	8	16042806	G	A	1106C>T	Thr369Ile	AT4G29990	Leucine-rich repeat transmembrane protein kinase protein
GLYMA08G21140	GLYMA08G21140.2	8	16045155	T	C	133A>G	Asn45Asp	AT4G29990	Leucine-rich repeat transmembrane protein kinase protein
GLYMA08G21140	GLYMA08G21140.2	8	16045180	G	C	108C>G	Ser36Arg	AT4G29990	Leucine-rich repeat transmembrane protein kinase protein
GLYMA08G21171	GLYMA08G21171.1	8	16067543	T	C	119T>C	Val40Ala	AT4G29990	Leucine-rich repeat transmembrane protein kinase protein
GLYMA08G21171	GLYMA08G21171.1	8	16067584	G	A	160G>A	Val54Ile	AT4G29990	Leucine-rich repeat transmembrane protein kinase protein
GLYMA08G21171	GLYMA08G21171.1	8	16067738	T	G	314T>G	Ile105Arg	AT4G29990	Leucine-rich repeat transmembrane protein kinase protein
GLYMA08G21171	GLYMA08G21171.1	8	16068156	G	C	598G>C	Val200Leu	AT4G29990	Leucine-rich repeat transmembrane protein kinase protein
GLYMA08G42971	GLYMA08G42971.1	8	42893699	C	T	925G>A	Glu309Lys	AT3G07040	NB-ARC domain-containing disease resistance protein
GLYMA08G26950	GLYMA08G26950.2	8	21285012	A	C	88T>G	Phe30Val	AT4G00660	RNAhelicase-like 8
GLYMA08G41544	GLYMA08G41544.1	8	41508254	C	A	1638G>T	Leu546Phe	AT5G45520	Leucine-rich repeat (LRR) family protein
GLYMA08G41544	GLYMA08G41544.1	8	41508355	T	C	1537A>G	Lys513Glu	AT5G45520	Leucine-rich repeat (LRR) family protein
GLYMA08G42971	GLYMA08G42971.1	8	42893557	C	T	1067G>A	Gly356Asp	AT3G07040	NB-ARC domain-containing disease resistance protein
GLYMA08G42971	GLYMA08G42971.1	8	42893680	C	G	944G>C	Arg315Thr	AT3G07040	NB-ARC domain-containing disease

									resistance protein
GLYMA08G42971	GLYMA08G42971.1	8	42894202	G	C	422C>G	Thr141Ser	AT3G07040	NB-ARC domain-containing disease resistance protein
GLYMA08G42971	GLYMA08G42971.1	8	42893740	A	T	884T>A	Val295Asp	AT3G07040	NB-ARC domain-containing disease resistance protein
GLYMA08G42971	GLYMA08G42971.1	8	42893741	C	T	883G>A	Val295Ile	AT3G07040	NB-ARC domain-containing disease resistance protein
GLYMA08G42971	GLYMA08G42971.1	8	42893754	A	T	870T>A	Phe290Leu	AT3G07040	NB-ARC domain-containing disease resistance protein
GLYMA08G42971	GLYMA08G42971.1	8	42893758	T	C	866A>G	Lys289Arg	AT3G07040	NB-ARC domain-containing disease resistance protein
GLYMA08G42971	GLYMA08G42971.1	8	42893777	T	C	847A>G	Asn283Asp	AT3G07040	NB-ARC domain-containing disease resistance protein
GLYMA08G42971	GLYMA08G42971.1	8	42893917	A	C	707T>G	Ile236Arg	AT3G07040	NB-ARC domain-containing disease resistance protein
GLYMA08G42971	GLYMA08G42971.1	8	42893957	G	T	667C>A	Pro223Thr	AT3G07040	NB-ARC domain-containing disease resistance protein
GLYMA08G42971	GLYMA08G42971.1	8	42894050	T	C	574A>G	Lys192Glu	AT3G07040	NB-ARC domain-containing disease resistance protein
GLYMA08G42971	GLYMA08G42971.1	8	42894061	T	A	563A>T	Lys188Ile	AT3G07040	NB-ARC domain-containing disease resistance protein
GLYMA08G42971	GLYMA08G42971.1	8	42894083	G	C	541C>G	His181Asp	AT3G07040	NB-ARC domain-containing disease resistance protein
GLYMA08G42971	GLYMA08G42971.1	8	42894128	G	T	496C>A	Leu166Met	AT3G07040	NB-ARC domain-containing disease resistance protein
GLYMA08G42971	GLYMA08G42971.1	8	42894166	T	G	458A>C	Asn153Thr	AT3G07040	NB-ARC domain-containing disease resistance protein
GLYMA08G42971	GLYMA08G42971.1	8	42894167	T	G	457A>C	Asn153His	AT3G07040	NB-ARC domain-containing disease resistance protein

GLYMA08G42971	GLYMA08G42971.2	8	42893699	C	T	925G>A	Glu309Lys	AT3G07040	NB-ARC domain-containing disease resistance protein
GLYMA08G42971	GLYMA08G42971.2	8	42893557	C	T	1067G>A	Gly356Asp	AT3G07040	NB-ARC domain-containing disease resistance protein
GLYMA08G42971	GLYMA08G42971.2	8	42893680	C	G	944G>C	Arg315Thr	AT3G07040	NB-ARC domain-containing disease resistance protein
GLYMA08G42971	GLYMA08G42971.2	8	42894202	G	C	422C>G	Thr141Ser	AT3G07040	NB-ARC domain-containing disease resistance protein
GLYMA08G42971	GLYMA08G42971.2	8	42893740	A	T	884T>A	Val295Asp	AT3G07040	NB-ARC domain-containing disease resistance protein
GLYMA08G42971	GLYMA08G42971.2	8	42893741	C	T	883G>A	Val295Ile	AT3G07040	NB-ARC domain-containing disease resistance protein
GLYMA08G42971	GLYMA08G42971.2	8	42893754	A	T	870T>A	Phe290Leu	AT3G07040	NB-ARC domain-containing disease resistance protein
GLYMA08G42971	GLYMA08G42971.2	8	42893758	T	C	866A>G	Lys289Arg	AT3G07040	NB-ARC domain-containing disease resistance protein
GLYMA08G42971	GLYMA08G42971.2	8	42893777	T	C	847A>G	Asn283Asp	AT3G07040	NB-ARC domain-containing disease resistance protein
GLYMA08G42971	GLYMA08G42971.2	8	42893917	A	C	707T>G	Ile236Arg	AT3G07040	NB-ARC domain-containing disease resistance protein
GLYMA08G42971	GLYMA08G42971.2	8	42893957	G	T	667C>A	Pro223Thr	AT3G07040	NB-ARC domain-containing disease resistance protein
GLYMA08G42971	GLYMA08G42971.2	8	42894050	T	C	574A>G	Lys192Glu	AT3G07040	NB-ARC domain-containing disease resistance protein
GLYMA08G42971	GLYMA08G42971.2	8	42894061	T	A	563A>T	Lys188Ile	AT3G07040	NB-ARC domain-containing disease resistance protein
GLYMA08G42971	GLYMA08G42971.2	8	42894083	G	C	541C>G	His181Asp	AT3G07040	NB-ARC domain-containing disease resistance protein
GLYMA08G42971	GLYMA08G42971.2	8	42894128	G	T	496C>A	Leu166Met	AT3G07040	NB-ARC domain-containing disease

									resistance protein
GLYMA08G42971	GLYMA08G42971.2	8	42894166	T	G	458A>C	Asn153Thr	AT3G07040	NB-ARC domain-containing disease resistance protein
GLYMA08G42971	GLYMA08G42971.2	8	42894167	T	G	457A>C	Asn153His	AT3G07040	NB-ARC domain-containing disease resistance protein
GLYMA08G26950	GLYMA08G26950.2	8	21284990	T	C	110A>G	His37Arg	AT4G00660	RNAhelicase-like 8
GLYMA09G08730	GLYMA09G08730.2	9	8141472	T	C	224T>C	Val75Ala	AT3G25620	ABC-2 type transporter family protein
GLYMA09G08730	GLYMA09G08730.2	9	8145622	G	T	1614G>T	Gln538His	AT3G25620	ABC-2 type transporter family protein
GLYMA09G08730	GLYMA09G08730.2	9	8145668	A	G	1660A>G	Arg554Gly	AT3G25620	ABC-2 type transporter family protein
GLYMA09G09597	GLYMA09G09597.1	9	9421496	G	T	367G>T	Val123Leu	AT3G44190	FAD/NAD(P)-binding oxidoreductase family protein
GLYMA09G09597	GLYMA09G09597.2	9	9421496	G	T	367G>T	Val123Leu	AT3G44190	FAD/NAD(P)-binding oxidoreductase family protein
GLYMA09G27827	GLYMA09G27827.1	9	34783134	T	C	61A>G	Thr21Ala	AT5G27610	DIRP ;Myb-like DNA-binding domain
GLYMA09G27827	GLYMA09G27827.1	9	34783139	A	T	56T>A	Val19Glu	AT5G27610	DIRP ;Myb-like DNA-binding domain
GLYMA09G27827	GLYMA09G27827.1	9	34783167	T	C	28A>G	Ile10Val	AT5G27610	DIRP ;Myb-like DNA-binding domain
GLYMA09G27827	GLYMA09G27827.1	9	34783187	T	G	8A>C	His3Pro	AT5G27610	DIRP ;Myb-like DNA-binding domain
GLYMA09G29970	GLYMA09G29970.1	9	36837807	C	T	380C>T	Ser127Leu	AT1G73460	Protein kinase superfamily protein
GLYMA09G29970	GLYMA09G29970.1	9	36839914	A	C	1744A>C	Met582Leu	AT1G73460	Protein kinase superfamily protein
GLYMA09G29970	GLYMA09G29970.1	9	36839916	G	A	1746G>A	Met582Ile	AT1G73460	Protein kinase superfamily protein
GLYMA09G41430	GLYMA09G41430.1	9	46107058	C	G	397C>G	Leu133Val	AT3G51650	N/A
GLYMA09G41430	GLYMA09G41430.1	9	46107168	T	G	507T>G	His169Gln	AT3G51650	N/A
GLYMA09G41430	GLYMA09G41430.1	9	46107339	T	G	678T>G	Phe226Leu	AT3G51650	N/A
GLYMA09G41430	GLYMA09G41430.1	9	46108123	G	T	1113G>T	Gln371His	AT3G51650	N/A

GLYMA09G41430	GLYMA09G41430.1	9	46108287	G	A	1277G>A	Ser426Asn	AT3G51650	N/A
GLYMA10G00230	GLYMA10G00230.1	10	39674	T	C	77T>C	Phe26Ser	N/A	N/A
GLYMA10G11635	GLYMA10G11635.1	10	11858339	T	C	188T>C	Phe63Ser	AT4G15020	hAT transposon superfamily
GLYMA10G11635	GLYMA10G11635.1	10	11858653	A	G	436A>G	Ser146Gly	AT4G15020	hAT transposon superfamily
GLYMA10G11635	GLYMA10G11635.1	10	11858923	C	T	631C>T	Pro211Ser	AT4G15020	hAT transposon superfamily
GLYMA10G11635	GLYMA10G11635.1	10	11859221	C	G	929C>G	Thr310Ser	AT4G15020	hAT transposon superfamily
GLYMA10G11635	GLYMA10G11635.1	10	11859384	G	T	959G>T	Cys320Phe	AT4G15020	hAT transposon superfamily
GLYMA10G11635	GLYMA10G11635.1	10	11859473	A	G	1048A>G	Met350Val	AT4G15020	hAT transposon superfamily
GLYMA10G11635	GLYMA10G11635.1	10	11859566	G	A	1141G>A	Ala381Thr	AT4G15020	hAT transposon superfamily
GLYMA10G11635	GLYMA10G11635.1	10	11859567	C	T	1142C>T	Ala381Val	AT4G15020	hAT transposon superfamily
GLYMA10G11635	GLYMA10G11635.1	10	11859593	A	G	1168A>G	Thr390Ala	AT4G15020	hAT transposon superfamily
GLYMA10G11635	GLYMA10G11635.1	10	11859981	A	G	1396A>G	Thr466Ala	AT4G15020	hAT transposon superfamily
GLYMA10G11635	GLYMA10G11635.1	10	11859999	T	G	1414T>G	Tyr472Asp	AT4G15020	hAT transposon superfamily
GLYMA10G11635	GLYMA10G11635.1	10	11860003	G	A	1418G>A	Ser473Asn	AT4G15020	hAT transposon superfamily
GLYMA10G11635	GLYMA10G11635.1	10	11860230	A	G	1552A>G	Lys518Glu	AT4G15020	hAT transposon superfamily
GLYMA10G12760	GLYMA10G12760.1	10	14289274	T	A	57T>A	Asp19Glu	AT5G56030	heat shock protein 81-2
GLYMA10G12760	GLYMA10G12760.1	10	14289330	G	T	113G>T	Gly38Val	AT5G56030	heat shock protein 81-2
GLYMA10G12760	GLYMA10G12760.1	10	14289368	G	A	151G>A	Glu51Lys	AT5G56030	heat shock protein 81-2
GLYMA10G12760	GLYMA10G12760.1	10	14289690	C	A	473C>A	Pro158His	AT5G56030	heat shock protein 81-2
GLYMA10G12760	GLYMA10G12760.1	10	14289728	G	C	511G>C	Val171Leu	AT5G56030	heat shock protein 81-2
GLYMA10G12771	GLYMA10G12771.1	10	14296108	T	C	445A>G	Ile149Val	N/A	N/A
GLYMA10G12771	GLYMA10G12771.1	10	14297322	T	C	250A>G	Arg84Gly	N/A	N/A
GLYMA10G37300	GLYMA10G37300.2	10	45309379	A	T	591A>T	Gln197His	AT2G34930	disease resistance family protein / LRR

									family protein
GLYMA10G37300	GLYMA10G37300.2	10	45311349	T	G	2561T>G	Leu854Arg	AT2G34930	disease resistance family protein / LRR family protein
GLYMA10G37300	GLYMA10G37300.2	10	45311379	G	A	2591G>A	Cys864Tyr	AT2G34930	disease resistance family protein / LRR family protein
GLYMA10G37300	GLYMA10G37300.2	10	45311400	T	A	2612T>A	Met871Lys	AT2G34930	disease resistance family protein / LRR family protein
GLYMA11G20830	GLYMA11G20830.1	11	17581373	T	A	703T>A	Ser235Thr	AT1G79150	binding
GLYMA11G18011	GLYMA11G18011.1	11	14476834	G	C	535G>C	Asp179His	AT3G49601	N/A
GLYMA11G18011	GLYMA11G18011.1	11	14476886	A	T	587A>T	Gln196Leu	AT3G49601	N/A
GLYMA11G18011	GLYMA11G18011.1	11	14477533	A	G	1061A>G	His354Arg	AT3G49601	N/A
GLYMA11G18011	GLYMA11G18011.1	11	14478413	A	G	1867A>G	Asn623Asp	AT3G49601	N/A
GLYMA11G18011	GLYMA11G18011.1	11	14478636	G	C	2090G>C	Gly697Ala	AT3G49601	N/A
GLYMA11G20830	GLYMA11G20830.1	11	17581086	C	T	551C>T	Ala184Val	AT1G79150	binding
GLYMA11G18011	GLYMA11G18011.1	11	14476887	A	T	588A>T	Gln196His	AT3G49601	N/A
GLYMA11G35880	GLYMA11G35880.1	11	37461930	C	T	290G>A	Ser97Asn	ATMG00990	NADH dehydrogenase 3
GLYMA11G35880	GLYMA11G35880.1	11	37462192	C	G	28G>C	Gly10Arg	ATMG00990	NADH dehydrogenase 3
GLYMA12G13345	GLYMA12G13345.1	12	11741785	C	T	553G>A	Glu185Lys	N/A	N/A
GLYMA12G13345	GLYMA12G13345.1	12	11741800	G	T	538C>A	Gln180Lys	N/A	N/A
GLYMA12G13345	GLYMA12G13345.1	12	11742114	C	T	224G>A	Arg75Gln	N/A	N/A
GLYMA12G13345	GLYMA12G13345.1	12	11742132	G	A	206C>T	Ala69Val	N/A	N/A
GLYMA13G05760	GLYMA13G05760.1	13	6092557	A	C	1756T>G	Ser586Ala	AT1G61040	plus-3 domain-containing protein
GLYMA13G05760	GLYMA13G05760.1	13	6092565	C	T	1748G>A	Arg583Lys	AT1G61040	plus-3 domain-containing protein
GLYMA13G05760	GLYMA13G05760.1	13	6092601	G	T	1712C>A	Pro571Gln	AT1G61040	plus-3 domain-containing protein
GLYMA13G05760	GLYMA13G05760.1	13	6092613	T	A	1700A>T	Tyr567Phe	AT1G61040	plus-3 domain-containing protein
GLYMA13G05760	GLYMA13G05760.1	13	6092832	G	A	1481C>T	Ala494Val	AT1G61040	plus-3 domain-containing protein
GLYMA13G05760	GLYMA13G05760.1	13	6092904	A	G	1409T>C	Ile470Thr	AT1G61040	plus-3 domain-containing protein

GLYMA13G05760	GLYMA13G05760.1	13	6092920	A	T	1393T>A	Leu465Met	AT1G61040	plus-3 domain-containing protein
GLYMA13G06151	GLYMA13G06151.1	13	6454089	G	C	619C>G	Gln207Glu	AT3G02100	UDP-Glycosyltransferase superfamily protein
GLYMA13G06151	GLYMA13G06151.1	13	6454139	A	C	569T>G	Leu190Trp	AT3G02100	UDP-Glycosyltransferase superfamily protein
GLYMA13G09794	GLYMA13G09794.1	13	11391677	A	C	493A>C	Lys165Gln	AT5G38260	Protein kinase superfamily protein
GLYMA13G09794	GLYMA13G09794.1	13	11395726	C	G	1817C>G	Thr606Arg	AT5G38260	Protein kinase superfamily protein
GLYMA13G09794	GLYMA13G09794.1	13	11395821	G	A	1912G>A	Val638Met	AT5G38260	Protein kinase superfamily protein
GLYMA13G09820	GLYMA13G09820.1	13	11413573	G	T	747G>T	Met249Ile	AT4G18250	receptor serine/threonine kinase, putative
GLYMA13G09820	GLYMA13G09820.1	13	11413637	G	A	811G>A	Val271Met	AT4G18250	receptor serine/threonine kinase, putative
GLYMA13G09840	GLYMA13G09840.2	13	11424198	C	T	1837G>A	Glu613Lys	AT1G66910	Protein kinase superfamily protein
GLYMA13G09840	GLYMA13G09840.2	13	11426196	T	A	36A>T	Leu12Phe	AT1G66910	Protein kinase superfamily protein
GLYMA13G30650	GLYMA13G30650.2	13	33198031	A	G	1145A>G	Asn382Ser	AT2G29120	glutamate receptor 2.7
GLYMA13G30650	GLYMA13G30650.2	13	33200584	A	T	2440A>T	Ser814Cys	AT2G29120	glutamate receptor 2.7
GLYMA13G30650	GLYMA13G30650.3	13	33198031	A	G	959A>G	Asn320Ser	AT2G29120	glutamate receptor 2.7
GLYMA13G30650	GLYMA13G30650.3	13	33200584	A	T	2254A>T	Ser752Cys	AT2G29120	glutamate receptor 2.7
GLYMA13G32270	GLYMA13G32270.1	13	34491678	A	T	1466T>A	Leu489His	AT4G21380	receptor kinase 3
GLYMA13G32270	GLYMA13G32270.1	13	34491684	G	A	1460C>T	Ser487Leu	AT4G21380	receptor kinase 3
GLYMA13G32270	GLYMA13G32270.2	13	34491678	A	T	1331T>A	Leu444His	AT4G21380	receptor kinase 3
GLYMA13G32270	GLYMA13G32270.2	13	34491684	G	A	1325C>T	Ser442Leu	AT4G21380	receptor kinase 3
GLYMA13G32270	GLYMA13G32270.3	13	34491678	A	T	1466T>A	Leu489His	AT4G21380	receptor kinase 3
GLYMA13G32270	GLYMA13G32270.3	13	34491684	G	A	1460C>T	Ser487Leu	AT4G21380	receptor kinase 3
GLYMA13G32270	GLYMA13G32270.4	13	34491678	A	T	1331T>A	Leu444His	AT4G21380	receptor kinase 3
GLYMA13G32270	GLYMA13G32270.4	13	34491684	G	A	1325C>T	Ser442Leu	AT4G21380	receptor kinase 3
GLYMA13G41440	GLYMA13G41440.2	13	41731981	T	C	2494T>C	Tyr832His	AT2G29510	Protein of unknown function (DUF3527)

GLYMA13G41440	GLYMA13G41440.2	13	41732011	C	A	2524C>A	Leu842Met	AT2G29510	Protein of unknown function (DUF3527)
GLYMA13G41440	GLYMA13G41440.3	13	41731981	T	C	2494T>C	Tyr832His	AT2G29510	Protein of unknown function (DUF3527)
GLYMA13G41440	GLYMA13G41440.3	13	41732011	C	A	2524C>A	Leu842Met	AT2G29510	Protein of unknown function (DUF3527)
GLYMA13G41440	GLYMA13G41440.4	13	41731981	T	C	2494T>C	Tyr832His	AT2G29510	Protein of unknown function (DUF3527)
GLYMA13G41440	GLYMA13G41440.4	13	41732011	C	A	2524C>A	Leu842Met	AT2G29510	Protein of unknown function (DUF3527)
GLYMA13G43503	GLYMA13G43503.1	13	43181270	G	C	465C>G	Phe155Leu	AT1G04390	BTB/POZ domain-containing protein
GLYMA13G43503	GLYMA13G43503.1	13	43181275	T	C	460A>G	Arg154Gly	AT1G04390	BTB/POZ domain-containing protein
GLYMA13G43503	GLYMA13G43503.1	13	43181296	G	T	439C>A	Leu147Met	AT1G04390	BTB/POZ domain-containing protein
GLYMA13G43503	GLYMA13G43503.1	13	43181312	A	C	423T>G	Phe141Leu	AT1G04390	BTB/POZ domain-containing protein
GLYMA13G43503	GLYMA13G43503.2	13	43181270	G	C	465C>G	Phe155Leu	AT1G04390	BTB/POZ domain-containing protein
GLYMA13G43503	GLYMA13G43503.2	13	43181275	T	C	460A>G	Arg154Gly	AT1G04390	BTB/POZ domain-containing protein
GLYMA13G43503	GLYMA13G43503.2	13	43181296	G	T	439C>A	Leu147Met	AT1G04390	BTB/POZ domain-containing protein
GLYMA13G43503	GLYMA13G43503.2	13	43181312	A	C	423T>G	Phe141Leu	AT1G04390	BTB/POZ domain-containing protein
GLYMA13G43503	GLYMA13G43503.3	13	43181270	G	C	393C>G	Phe131Leu	AT1G04390	BTB/POZ domain-containing protein
GLYMA13G43503	GLYMA13G43503.3	13	43181275	T	C	388A>G	Arg130Gly	AT1G04390	BTB/POZ domain-containing protein
GLYMA13G43503	GLYMA13G43503.3	13	43181296	G	T	367C>A	Leu123Met	AT1G04390	BTB/POZ domain-containing protein
GLYMA13G43503	GLYMA13G43503.3	13	43181312	A	C	351T>G	Phe117Leu	AT1G04390	BTB/POZ domain-containing protein
GLYMA13G43503	GLYMA13G43503.4	13	43181270	G	C	393C>G	Phe131Leu	AT1G04390	BTB/POZ domain-containing protein
GLYMA13G43503	GLYMA13G43503.4	13	43181275	T	C	388A>G	Arg130Gly	AT1G04390	BTB/POZ domain-containing protein
GLYMA13G43503	GLYMA13G43503.4	13	43181296	G	T	367C>A	Leu123Met	AT1G04390	BTB/POZ domain-containing protein
GLYMA13G43503	GLYMA13G43503.4	13	43181312	A	C	351T>G	Phe117Leu	AT1G04390	BTB/POZ domain-containing protein
GLYMA14G22793	GLYMA14G22793.1	14	26958753	G	A	1313G>A	Ser438Asn	N/A	N/A

GLYMA14G22793	GLYMA14G22793.1	14	26958828	G	A	1388G>A	Ser463Asn	N/A	N/A
GLYMA14G22793	GLYMA14G22793.1	14	26959022	A	G	1582A>G	Thr528Ala	N/A	N/A
GLYMA14G22793	GLYMA14G22793.1	14	26959035	C	T	1595C>T	Thr532Ile	N/A	N/A
GLYMA14G22793	GLYMA14G22793.1	14	26959173	C	A	1733C>A	Thr578Asn	N/A	N/A
GLYMA14G22793	GLYMA14G22793.1	14	26959238	C	A	1798C>A	His600Asn	N/A	N/A
GLYMA14G22793	GLYMA14G22793.1	14	26959307	A	G	1867A>G	Ser623Gly	N/A	N/A
GLYMA14G28005	GLYMA14G28005.1	14	34356035	C	G	696G>C	Leu232Phe	AT1G78810	N/A
GLYMA14G28005	GLYMA14G28005.1	14	34356067	C	T	664G>A	Ala222Thr	AT1G78810	N/A
GLYMA14G28005	GLYMA14G28005.1	14	34356087	C	G	644G>C	Trp215Ser	AT1G78810	N/A
GLYMA14G28005	GLYMA14G28005.1	14	34356117	C	T	614G>A	Cys205Tyr	AT1G78810	N/A
GLYMA14G28005	GLYMA14G28005.1	14	34356139	T	C	592A>G	Arg198Gly	AT1G78810	N/A
GLYMA14G28005	GLYMA14G28005.1	14	34356178	T	A	553A>T	Thr185Ser	AT1G78810	N/A
GLYMA14G28005	GLYMA14G28005.1	14	34356214	T	C	517A>G	Arg173Gly	AT1G78810	N/A
GLYMA15G19280	GLYMA15G19280.1	15	16522916	T	G	97T>G	Leu33Val	AT4G25440	zinc finger WD40 repeat protein 1
GLYMA15G19280	GLYMA15G19280.1	15	16523042	A	G	223A>G	Arg75Gly	AT4G25440	zinc finger WD40 repeat protein 1
GLYMA15G19290	GLYMA15G19290.2	15	16554102	G	A	71G>A	Arg24Lys	AT4G25440	zinc finger WD40 repeat protein 1
GLYMA15G22384	GLYMA15G22384.1	15	20980146	G	C	2108C>G	Ser703Trp	AT2G34680	Outer arm dynein light chain 1 protein
GLYMA15G33721	GLYMA15G33721.1	15	37581700	C	T	934G>A	Asp312Asn	AT3G11760	N/A
GLYMA15G33721	GLYMA15G33721.1	15	37581703	G	A	931C>T	Arg311Cys	AT3G11760	N/A
GLYMA15G33721	GLYMA15G33721.1	15	37581783	G	A	851C>T	Ala284Val	AT3G11760	N/A
GLYMA15G33721	GLYMA15G33721.2	15	37581700	C	T	883G>A	Asp295Asn	AT3G11760	N/A
GLYMA15G33721	GLYMA15G33721.2	15	37581703	G	A	880C>T	Arg294Cys	AT3G11760	N/A
GLYMA15G33721	GLYMA15G33721.2	15	37581783	G	A	800C>T	Ala267Val	AT3G11760	N/A
GLYMA15G39090	GLYMA15G39090.2	15	45668524	A	T	449A>T	Lys150Met	AT3G14690	cytochrome P450, family 72, subfamily A, polypeptide 15
GLYMA15G39090	GLYMA15G39090.2	15	45669409	C	G	614C>G	Ala205Gly	AT3G14690	cytochrome P450, family 72, subfamily A, polypeptide 15
GLYMA15G39090	GLYMA15G39090.2	15	45669487	T	C	692T>C	Ile231Thr	AT3G14690	cytochrome P450, family 72, subfamily

									A, polypeptide 15
GLYMA15G39090	GLYMA15G39090.2	15	45669496	C	T	701C>T	Thr234Ile	AT3G14690	cytochrome P450, family 72, subfamily A, polypeptide 15
GLYMA15G39090	GLYMA15G39090.2	15	45669756	A	T	743A>T	Lys248Met	AT3G14690	cytochrome P450, family 72, subfamily A, polypeptide 15
GLYMA15G39090	GLYMA15G39090.4	15	45668524	A	T	449A>T	Lys150Met	AT3G14690	cytochrome P450, family 72, subfamily A, polypeptide 15
GLYMA15G39090	GLYMA15G39090.4	15	45669409	C	G	614C>G	Ala205Gly	AT3G14690	cytochrome P450, family 72, subfamily A, polypeptide 15
GLYMA15G39090	GLYMA15G39090.4	15	45669487	T	C	692T>C	Ile231Thr	AT3G14690	cytochrome P450, family 72, subfamily A, polypeptide 15
GLYMA15G39090	GLYMA15G39090.4	15	45669496	C	T	701C>T	Thr234Ile	AT3G14690	cytochrome P450, family 72, subfamily A, polypeptide 15
GLYMA15G39090	GLYMA15G39090.4	15	45669756	A	T	743A>T	Lys248Met	AT3G14690	cytochrome P450, family 72, subfamily A, polypeptide 15
GLYMA15G39090	GLYMA15G39090.5	15	45668524	A	T	449A>T	Lys150Met	AT3G14690	cytochrome P450, family 72, subfamily A, polypeptide 15
GLYMA15G39090	GLYMA15G39090.5	15	45669409	C	G	614C>G	Ala205Gly	AT3G14690	cytochrome P450, family 72, subfamily A, polypeptide 15
GLYMA15G39090	GLYMA15G39090.5	15	45669487	T	C	692T>C	Ile231Thr	AT3G14690	cytochrome P450, family 72, subfamily A, polypeptide 15
GLYMA15G39090	GLYMA15G39090.5	15	45669496	C	T	701C>T	Thr234Ile	AT3G14690	cytochrome P450, family 72, subfamily A, polypeptide 15
GLYMA15G39090	GLYMA15G39090.5	15	45669756	A	T	743A>T	Lys248Met	AT3G14690	cytochrome P450, family 72, subfamily A, polypeptide 15
GLYMA15G39090	GLYMA15G39090.7	15	45668524	A	T	65A>T	Lys22Met	AT3G14690	cytochrome P450, family 72, subfamily A, polypeptide 15
GLYMA15G39090	GLYMA15G39090.7	15	45669409	C	G	230C>G	Ala77Gly	AT3G14690	cytochrome P450, family 72, subfamily A, polypeptide 15

GLYMA15G39090	GLYMA15G39090.7	15	45669487	T	C	308T>C	Ile103Thr	AT3G14690	cytochrome P450, family 72, subfamily A, polypeptide 15
GLYMA15G39090	GLYMA15G39090.7	15	45669496	C	T	317C>T	Thr106Ile	AT3G14690	cytochrome P450, family 72, subfamily A, polypeptide 15
GLYMA15G39090	GLYMA15G39090.7	15	45669756	A	T	359A>T	Lys120Met	AT3G14690	cytochrome P450, family 72, subfamily A, polypeptide 15
GLYMA15G39642	GLYMA15G39642.1	15	46459249	C	T	89C>T	Ser30Phe	AT4G27190	NB-ARC domain-containing disease resistance protein
GLYMA15G39642	GLYMA15G39642.1	15	46460529	G	A	1369G>A	Glu457Lys	AT4G27190	NB-ARC domain-containing disease resistance protein
GLYMA15G39642	GLYMA15G39642.1	15	46460602	T	C	1442T>C	Ile481Thr	AT4G27190	NB-ARC domain-containing disease resistance protein
GLYMA15G39642	GLYMA15G39642.1	15	46460731	G	T	1571G>T	Ser524Ile	AT4G27190	NB-ARC domain-containing disease resistance protein
GLYMA15G39642	GLYMA15G39642.1	15	46460739	G	A	1579G>A	Glu527Lys	AT4G27190	NB-ARC domain-containing disease resistance protein
GLYMA15G39642	GLYMA15G39642.1	15	46460766	C	T	1606C>T	Pro536Ser	AT4G27190	NB-ARC domain-containing disease resistance protein
GLYMA15G39642	GLYMA15G39642.1	15	46460783	G	C	1623G>C	Leu541Phe	AT4G27190	NB-ARC domain-containing disease resistance protein
GLYMA15G39642	GLYMA15G39642.1	15	46460789	G	C	1629G>C	Lys543Asn	AT4G27190	NB-ARC domain-containing disease resistance protein
GLYMA15G39642	GLYMA15G39642.1	15	46460814	A	C	1654A>C	Lys552Gln	AT4G27190	NB-ARC domain-containing disease resistance protein
GLYMA15G39642	GLYMA15G39642.1	15	46460875	A	G	1715A>G	Glu572Gly	AT4G27190	NB-ARC domain-containing disease resistance protein
GLYMA15G39642	GLYMA15G39642.2	15	46459249	C	T	89C>T	Ser30Phe	AT4G27190	NB-ARC domain-containing disease resistance protein
GLYMA15G39642	GLYMA15G39642.2	15	46460529	G	A	1369G>A	Glu457Lys	AT4G27190	NB-ARC domain-containing disease

									resistance protein
GLYMA15G39642	GLYMA15G39642.2	15	46460602	T	C	1442T>C	Ile481Thr	AT4G27190	NB-ARC domain-containing disease resistance protein
GLYMA15G39642	GLYMA15G39642.2	15	46460731	G	T	1571G>T	Ser524Ile	AT4G27190	NB-ARC domain-containing disease resistance protein
GLYMA15G39642	GLYMA15G39642.2	15	46460739	G	A	1579G>A	Glu527Lys	AT4G27190	NB-ARC domain-containing disease resistance protein
GLYMA15G39642	GLYMA15G39642.2	15	46460766	C	T	1606C>T	Pro536Ser	AT4G27190	NB-ARC domain-containing disease resistance protein
GLYMA15G39642	GLYMA15G39642.2	15	46460783	G	C	1623G>C	Leu541Phe	AT4G27190	NB-ARC domain-containing disease resistance protein
GLYMA15G39642	GLYMA15G39642.2	15	46460789	G	C	1629G>C	Lys543Asn	AT4G27190	NB-ARC domain-containing disease resistance protein
GLYMA15G39642	GLYMA15G39642.2	15	46460814	A	C	1654A>C	Lys552Gln	AT4G27190	NB-ARC domain-containing disease resistance protein
GLYMA15G39642	GLYMA15G39642.2	15	46460875	A	G	1715A>G	Glu572Gly	AT4G27190	NB-ARC domain-containing disease resistance protein
GLYMA15G39642	GLYMA15G39642.3	15	46459249	C	T	89C>T	Ser30Phe	AT4G27190	NB-ARC domain-containing disease resistance protein
GLYMA15G39642	GLYMA15G39642.3	15	46460529	G	A	1369G>A	Glu457Lys	AT4G27190	NB-ARC domain-containing disease resistance protein
GLYMA15G39642	GLYMA15G39642.3	15	46460602	T	C	1442T>C	Ile481Thr	AT4G27190	NB-ARC domain-containing disease resistance protein
GLYMA15G39642	GLYMA15G39642.3	15	46460731	G	T	1571G>T	Ser524Ile	AT4G27190	NB-ARC domain-containing disease resistance protein
GLYMA15G39642	GLYMA15G39642.3	15	46460739	G	A	1579G>A	Glu527Lys	AT4G27190	NB-ARC domain-containing disease resistance protein
GLYMA15G39642	GLYMA15G39642.3	15	46460766	C	T	1606C>T	Pro536Ser	AT4G27190	NB-ARC domain-containing disease resistance protein

GLYMA15G39642	GLYMA15G39642.3	15	46460783	G	C	1623G>C	Leu541Phe	AT4G27190	NB-ARC domain-containing disease resistance protein
GLYMA15G39642	GLYMA15G39642.3	15	46460789	G	C	1629G>C	Lys543Asn	AT4G27190	NB-ARC domain-containing disease resistance protein
GLYMA15G39642	GLYMA15G39642.3	15	46460814	A	C	1654A>C	Lys552Gln	AT4G27190	NB-ARC domain-containing disease resistance protein
GLYMA15G39642	GLYMA15G39642.3	15	46460875	A	G	1715A>G	Glu572Gly	AT4G27190	NB-ARC domain-containing disease resistance protein
GLYMA16G17441	GLYMA16G17441.1	16	18901339	T	A	152T>A	Val51Asp	AT2G34930	disease resistance family protein / LRR family protein
GLYMA16G21891	GLYMA16G21891.1	16	25230352	A	T	155A>T	Asn52Ile	ATCG00130	ATPase, F0 complex, subunit B/B', bacterial/chloroplast
GLYMA16G21891	GLYMA16G21891.1	16	25230358	T	C	161T>C	Met54Thr	ATCG00130	ATPase, F0 complex, subunit B/B', bacterial/chloroplast
GLYMA16G17441	GLYMA16G17441.1	16	18902761	T	C	1394T>C	Ile465Thr	AT2G34930	disease resistance family protein / LRR family protein
GLYMA16G17441	GLYMA16G17441.1	16	18902957	A	C	1505A>C	Glu502Ala	AT2G34930	disease resistance family protein / LRR family protein
GLYMA16G17441	GLYMA16G17441.1	16	18902995	C	A	1543C>A	Leu515Ile	AT2G34930	disease resistance family protein / LRR family protein
GLYMA16G17441	GLYMA16G17441.1	16	18903105	G	A	1653G>A	Met551Ile	AT2G34930	disease resistance family protein / LRR family protein
GLYMA16G31331	GLYMA16G31331.1	16	34737771	C	T	1421C>T	Ala474Val	AT5G52300	CAP160 protein
GLYMA16G17441	GLYMA16G17441.1	16	18903605	A	T	1976A>T	Lys659Ile	AT2G34930	disease resistance family protein / LRR family protein
GLYMA16G17441	GLYMA16G17441.1	16	18901819	A	T	452A>T	His151Leu	AT2G34930	disease resistance family protein / LRR family protein
GLYMA16G19790	GLYMA16G19790.2	16	22051927	A	G	379A>G	Thr127Ala	AT4G37990	elicitor-activated gene 3-2
GLYMA16G19790	GLYMA16G19790.2	16	22051939	A	C	391A>C	Ile131Leu	AT4G37990	elicitor-activated gene

									3-2
GLYMA16G17441	GLYMA16G17441.1	16	18901831	C	T	464C>T	Ser155Phe	AT2G34930	disease resistance family protein / LRR family protein
GLYMA16G17441	GLYMA16G17441.1	16	18903155	G	A	1703G>A	Gly568Glu	AT2G34930	disease resistance family protein / LRR family protein
GLYMA16G19790	GLYMA16G19790.2	16	22052150	A	G	602A>G	Asn201Ser	AT4G37990	elicitor-activated gene 3-2
GLYMA16G21891	GLYMA16G21891.1	16	25230240	A	C	43A>C	Lys15Gln	ATCG00130	ATPase, F0 complex, subunit B/B', bacterial/chloroplast
GLYMA16G21891	GLYMA16G21891.1	16	25230262	A	G	65A>G	His22Arg	ATCG00130	ATPase, F0 complex, subunit B/B', bacterial/chloroplast
GLYMA16G21891	GLYMA16G21891.1	16	25230297	T	C	100T>C	Trp34Arg	ATCG00130	ATPase, F0 complex, subunit B/B', bacterial/chloroplast
GLYMA16G17441	GLYMA16G17441.1	16	18903849	G	A	2122G>A	Ala708Thr	AT2G34930	disease resistance family protein / LRR family protein
GLYMA16G23500	GLYMA16G23500.2	16	27259589	T	G	706T>G	Ser236Ala	AT2G34930	disease resistance family protein / LRR family protein
GLYMA16G21891	GLYMA16G21891.1	16	25230517	C	T	320C>T	Ala107Val	ATCG00130	ATPase, F0 complex, subunit B/B', bacterial/chloroplast
GLYMA16G23500	GLYMA16G23500.2	16	27259208	A	G	509A>G	Tyr170Cys	AT2G34930	disease resistance family protein / LRR family protein
GLYMA16G23500	GLYMA16G23500.2	16	27259547	A	C	664A>C	Asn222His	AT2G34930	disease resistance family protein / LRR family protein
GLYMA16G23500	GLYMA16G23500.2	16	27259604	C	A	721C>A	Pro241Thr	AT2G34930	disease resistance family protein / LRR family protein
GLYMA16G23500	GLYMA16G23500.2	16	27259649	T	A	766T>A	Leu256Met	AT2G34930	disease resistance family protein / LRR family protein
GLYMA16G28701	GLYMA16G28701.1	16	32646656	G	C	277C>G	Leu93Val	AT2G34930	disease resistance family protein / LRR family protein
GLYMA16G23500	GLYMA16G23500.2	16	27259652	A	T	769A>T	Thr257Ser	AT2G34930	disease resistance

									family protein / LRR family protein
GLYMA16G23790	GLYMA16G23790.3	16	27652196	C	G	196C>G	Gln66Glu	AT5G36930	Disease resistance protein (TIR-NBS-LRR class) family
GLYMA16G23790	GLYMA16G23790.3	16	27652232	G	C	232G>C	Asp78His	AT5G36930	Disease resistance protein (TIR-NBS-LRR class) family
GLYMA16G23790	GLYMA16G23790.3	16	27652277	C	T	277C>T	Leu93Phe	AT5G36930	Disease resistance protein (TIR-NBS-LRR class) family
GLYMA16G23790	GLYMA16G23790.3	16	27652341	G	A	341G>A	Arg114Lys	AT5G36930	Disease resistance protein (TIR-NBS-LRR class) family
GLYMA16G23790	GLYMA16G23790.3	16	27652448	A	G	448A>G	Asn150Asp	AT5G36930	Disease resistance protein (TIR-NBS-LRR class) family
GLYMA16G23790	GLYMA16G23790.3	16	27652714	G	C	526G>C	Gly176Arg	AT5G36930	Disease resistance protein (TIR-NBS-LRR class) family
GLYMA16G23790	GLYMA16G23790.3	16	27652729	G	C	541G>C	Gly181Arg	AT5G36930	Disease resistance protein (TIR-NBS-LRR class) family
GLYMA16G23790	GLYMA16G23790.3	16	27652733	C	T	545C>T	Pro182Leu	AT5G36930	Disease resistance protein (TIR-NBS-LRR class) family
GLYMA16G23790	GLYMA16G23790.3	16	27653573	G	A	1385G>A	Gly462Asp	AT5G36930	Disease resistance protein (TIR-NBS-LRR class) family
GLYMA16G23790	GLYMA16G23790.3	16	27653973	T	A	1619T>A	Leu540Gln	AT5G36930	Disease resistance protein (TIR-NBS-LRR class) family
GLYMA16G23790	GLYMA16G23790.3	16	27653982	C	G	1628C>G	Ser543Cys	AT5G36930	Disease resistance protein (TIR-NBS-LRR class) family
GLYMA16G23790	GLYMA16G23790.3	16	27653999	G	A	1645G>A	Ala549Thr	AT5G36930	Disease resistance protein (TIR-NBS-LRR class) family
GLYMA16G23790	GLYMA16G23790.3	16	27654002	A	G	1648A>G	Thr550Ala	AT5G36930	Disease resistance protein (TIR-NBS-LRR class) family
GLYMA16G23790	GLYMA16G23790.3	16	27654041	A	C	1687A>C	Asn563His	AT5G36930	Disease resistance protein (TIR-NBS-LRR class) family

GLYMA16G23790	GLYMA16G23790.3	16	27654069	G	T	1715G>T	Gly572Val	AT5G36930	Disease resistance protein (TIR-NBS-LRR class) family
GLYMA16G23790	GLYMA16G23790.3	16	27654104	A	G	1750A>G	Ser584Gly	AT5G36930	Disease resistance protein (TIR-NBS-LRR class) family
GLYMA16G23790	GLYMA16G23790.3	16	27654113	T	G	1759T>G	Leu587Val	AT5G36930	Disease resistance protein (TIR-NBS-LRR class) family
GLYMA16G23790	GLYMA16G23790.3	16	27654128	C	G	1774C>G	Arg592Gly	AT5G36930	Disease resistance protein (TIR-NBS-LRR class) family
GLYMA16G23790	GLYMA16G23790.3	16	27654177	C	T	1823C>T	Ala608Val	AT5G36930	Disease resistance protein (TIR-NBS-LRR class) family
GLYMA16G23790	GLYMA16G23790.3	16	27654216	G	A	1862G>A	Gly621Glu	AT5G36930	Disease resistance protein (TIR-NBS-LRR class) family
GLYMA16G23790	GLYMA16G23790.3	16	27654717	G	T	2261G>T	Gly754Val	AT5G36930	Disease resistance protein (TIR-NBS-LRR class) family
GLYMA16G23790	GLYMA16G23790.3	16	27654824	C	T	2368C>T	Arg790Cys	AT5G36930	Disease resistance protein (TIR-NBS-LRR class) family
GLYMA16G23790	GLYMA16G23790.3	16	27654878	A	G	2422A>G	Asn808Asp	AT5G36930	Disease resistance protein (TIR-NBS-LRR class) family
GLYMA16G23790	GLYMA16G23790.3	16	27654920	G	A	2464G>A	Val822Met	AT5G36930	Disease resistance protein (TIR-NBS-LRR class) family
GLYMA16G23790	GLYMA16G23790.3	16	27654929	G	T	2473G>T	Asp825Tyr	AT5G36930	Disease resistance protein (TIR-NBS-LRR class) family
GLYMA16G23790	GLYMA16G23790.3	16	27654997	G	T	2541G>T	Leu847Phe	AT5G36930	Disease resistance protein (TIR-NBS-LRR class) family
GLYMA16G23790	GLYMA16G23790.3	16	27655011	A	G	2555A>G	Lys852Arg	AT5G36930	Disease resistance protein (TIR-NBS-LRR class) family
GLYMA16G23790	GLYMA16G23790.3	16	27655012	A	T	2556A>T	Lys852Asn	AT5G36930	Disease resistance protein (TIR-NBS-LRR class) family
GLYMA16G23790	GLYMA16G23790.3	16	27655055	G	C	2599G>C	Val867Leu	AT5G36930	Disease resistance protein (TIR-NBS-

									LRR class) family
GLYMA16G23790	GLYMA16G23790.3	16	27655128	T	C	2672T>C	Leu891Ser	AT5G36930	Disease resistance protein (TIR-NBS-LRR class) family
GLYMA16G23790	GLYMA16G23790.3	16	27655300	A	T	2751A>T	Gln917His	AT5G36930	Disease resistance protein (TIR-NBS-LRR class) family
GLYMA16G23790	GLYMA16G23790.3	16	27655308	A	G	2759A>G	Glu920Gly	AT5G36930	Disease resistance protein (TIR-NBS-LRR class) family
GLYMA16G23790	GLYMA16G23790.3	16	27655317	T	G	2768T>G	Ile923Ser	AT5G36930	Disease resistance protein (TIR-NBS-LRR class) family
GLYMA16G23790	GLYMA16G23790.3	16	27655979	G	C	3280G>C	Glu1094Gln	AT5G36930	Disease resistance protein (TIR-NBS-LRR class) family
GLYMA16G33616	GLYMA16G33616.1	16	36481184	G	A	1306G>A	Glu436Lys	AT5G36930	Disease resistance protein (TIR-NBS-LRR class) family
GLYMA16G23800	GLYMA16G23800.2	16	27664722	G	T	2234C>A	Thr745Lys	AT5G17680	disease resistance protein (TIR-NBS-LRR class), putative
GLYMA16G23800	GLYMA16G23800.2	16	27664726	T	C	2230A>G	Lys744Glu	AT5G17680	disease resistance protein (TIR-NBS-LRR class), putative
GLYMA16G23800	GLYMA16G23800.2	16	27664823	A	C	2133T>G	Phe711Leu	AT5G17680	disease resistance protein (TIR-NBS-LRR class), putative
GLYMA16G33616	GLYMA16G33616.1	16	36482134	C	G	1978C>G	His660Asp	AT5G36930	Disease resistance protein (TIR-NBS-LRR class) family
GLYMA16G33616	GLYMA16G33616.1	16	36482200	G	A	2044G>A	Gly682Ser	AT5G36930	Disease resistance protein (TIR-NBS-LRR class) family
GLYMA16G33616	GLYMA16G33616.1	16	36482209	A	T	2053A>T	Arg685Trp	AT5G36930	Disease resistance protein (TIR-NBS-LRR class) family
GLYMA16G23800	GLYMA16G23800.2	16	27666533	C	T	694G>A	Ala232Thr	AT5G17680	disease resistance protein (TIR-NBS-LRR class), putative
GLYMA16G23800	GLYMA16G23800.2	16	27666548	T	G	679A>C	Ser227Arg	AT5G17680	disease resistance protein (TIR-NBS-LRR class), putative

GLYMA16G23800	GLYMA16G23800.2	16	27666814	G	A	413C>T	Pro138Leu	AT5G17680	disease resistance protein (TIR-NBS-LRR class), putative
GLYMA16G24920	GLYMA16G24920.2	16	28895254	A	T	1965T>A	Asn655Lys	AT5G17680	disease resistance protein (TIR-NBS-LRR class), putative
GLYMA16G24920	GLYMA16G24920.2	16	28895263	A	T	1956T>A	Asn652Lys	AT5G17680	disease resistance protein (TIR-NBS-LRR class), putative
GLYMA16G33616	GLYMA16G33616.1	16	36482217	G	C	2061G>C	Arg687Ser	AT5G36930	Disease resistance protein (TIR-NBS-LRR class) family
GLYMA16G24920	GLYMA16G24920.2	16	28895545	T	C	1790A>G	His597Arg	AT5G17680	disease resistance protein (TIR-NBS-LRR class), putative
GLYMA16G33616	GLYMA16G33616.1	16	36483623	A	T	3272A>T	Tyr1091Phe	AT5G36930	Disease resistance protein (TIR-NBS-LRR class) family
GLYMA16G33616	GLYMA16G33616.1	16	36484094	C	G	3743C>G	Ala1248Gly	AT5G36930	Disease resistance protein (TIR-NBS-LRR class) family
GLYMA16G33616	GLYMA16G33616.1	16	36484301	G	C	3950G>C	Arg1317Pro	AT5G36930	Disease resistance protein (TIR-NBS-LRR class) family
GLYMA16G27544	GLYMA16G27544.1	16	31559949	A	C	2380A>C	Thr794Pro	AT5G17680	disease resistance protein (TIR-NBS-LRR class), putative
GLYMA16G33616	GLYMA16G33616.1	16	36484309	C	G	3958C>G	Pro1320Ala	AT5G36930	Disease resistance protein (TIR-NBS-LRR class) family
GLYMA16G33616	GLYMA16G33616.1	16	36484313	A	C	3962A>C	Lys1321Thr	AT5G36930	Disease resistance protein (TIR-NBS-LRR class) family
GLYMA16G33616	GLYMA16G33616.1	16	36484328	A	C	3977A>C	His1326Pro	AT5G36930	Disease resistance protein (TIR-NBS-LRR class) family
GLYMA16G33616	GLYMA16G33616.1	16	36484332	A	T	3981A>T	Glu1327Asp	AT5G36930	Disease resistance protein (TIR-NBS-LRR class) family
GLYMA16G33616	GLYMA16G33616.2	16	36481184	G	A	1306G>A	Glu436Lys	AT5G36930	Disease resistance protein (TIR-NBS-LRR class) family
GLYMA16G33616	GLYMA16G33616.2	16	36482134	C	G	1978C>G	His660Asp	AT5G36930	Disease resistance protein (TIR-NBS-

									LRR class) family
GLYMA16G33616	GLYMA16G33616.2	16	36482200	G	A	2044G>A	Gly682Ser	AT5G36930	Disease resistance protein (TIR-NBS-LRR class) family
GLYMA16G28460	GLYMA16G28460.2	16	32411501	T	C	3023T>C	Leu1008Pro	AT2G15080	receptor like protein 19
GLYMA16G33616	GLYMA16G33616.2	16	36482209	A	T	2053A>T	Arg685Trp	AT5G36930	Disease resistance protein (TIR-NBS-LRR class) family
GLYMA16G33616	GLYMA16G33616.2	16	36482217	G	C	2061G>C	Arg687Ser	AT5G36930	Disease resistance protein (TIR-NBS-LRR class) family
GLYMA16G33616	GLYMA16G33616.2	16	36483623	A	T	3203A>T	Tyr1068Phe	AT5G36930	Disease resistance protein (TIR-NBS-LRR class) family
GLYMA16G33616	GLYMA16G33616.2	16	36484094	C	G	3674C>G	Ala1225Gly	AT5G36930	Disease resistance protein (TIR-NBS-LRR class) family
GLYMA16G33616	GLYMA16G33616.2	16	36484301	G	C	3881G>C	Arg1294Pro	AT5G36930	Disease resistance protein (TIR-NBS-LRR class) family
GLYMA16G33616	GLYMA16G33616.2	16	36484309	C	G	3889C>G	Pro1297Ala	AT5G36930	Disease resistance protein (TIR-NBS-LRR class) family
GLYMA16G28480	GLYMA16G28480.1	16	32422689	C	T	854C>T	Ser285Phe	AT2G15080	receptor like protein 19
GLYMA16G33616	GLYMA16G33616.2	16	36484313	A	C	3893A>C	Lys1298Thr	AT5G36930	Disease resistance protein (TIR-NBS-LRR class) family
GLYMA16G33616	GLYMA16G33616.2	16	36484328	A	C	3908A>C	His1303Pro	AT5G36930	Disease resistance protein (TIR-NBS-LRR class) family
GLYMA16G28480	GLYMA16G28480.1	16	32422896	G	A	1061G>A	Ser354Asn	AT2G15080	receptor like protein 19
GLYMA16G28480	GLYMA16G28480.1	16	32422982	G	C	1147G>C	Asp383His	AT2G15080	receptor like protein 19
GLYMA16G33616	GLYMA16G33616.2	16	36484332	A	T	3912A>T	Glu1304Asp	AT5G36930	Disease resistance protein (TIR-NBS-LRR class) family
GLYMA16G23800	GLYMA16G23800.2	16	27664591	A	T	2365T>A	Phe789Ile	AT5G17680	disease resistance protein (TIR-NBS-LRR class), putative
GLYMA16G28520	GLYMA16G28520.2	16	32450878	G	C	486G>C	Glu162Asp	AT3G05650	receptor like protein

									32
GLYMA16G28520	GLYMA16G28520.2	16	32451066	C	T	674C>T	Ser225Phe	AT3G05650	receptor like protein 32
GLYMA16G23800	GLYMA16G23800.2	16	27664867	G	A	2089C>T	Leu697Phe	AT5G17680	disease resistance protein (TIR-NBS-LRR class), putative
GLYMA16G28520	GLYMA16G28520.2	16	32452275	C	A	1883C>A	Thr628Lys	AT3G05650	receptor like protein 32
GLYMA16G28520	GLYMA16G28520.2	16	32452338	C	G	1946C>G	Thr649Ser	AT3G05650	receptor like protein 32
GLYMA16G28670	GLYMA16G28670.2	16	32612656	G	C	115G>C	Glu39Gln	AT2G34930	disease resistance family protein / LRR family protein
GLYMA16G28670	GLYMA16G28670.2	16	32613055	T	C	514T>C	Phe172Leu	AT2G34930	disease resistance family protein / LRR family protein
GLYMA16G28670	GLYMA16G28670.2	16	32614297	A	T	1756A>T	Ile586Phe	AT2G34930	disease resistance family protein / LRR family protein
GLYMA16G28670	GLYMA16G28670.2	16	32614313	A	G	1772A>G	Gln591Arg	AT2G34930	disease resistance family protein / LRR family protein
GLYMA16G28670	GLYMA16G28670.2	16	32614331	G	A	1790G>A	Ser597Asn	AT2G34930	disease resistance family protein / LRR family protein
GLYMA16G28701	GLYMA16G28701.1	16	32645794	T	G	662A>C	Asn221Thr	AT2G34930	disease resistance family protein / LRR family protein
GLYMA16G28701	GLYMA16G28701.1	16	32646032	T	G	424A>C	Ile142Leu	AT2G34930	disease resistance family protein / LRR family protein
GLYMA16G23800	GLYMA16G23800.2	16	27664875	T	A	2081A>T	His694Leu	AT5G17680	disease resistance protein (TIR-NBS-LRR class), putative
GLYMA16G28701	GLYMA16G28701.1	16	32646932	G	C	1C>G	Leu1Val	AT2G34930	disease resistance family protein / LRR family protein
GLYMA16G31331	GLYMA16G31331.1	16	34737026	T	A	676T>A	Ser226Thr	AT5G52300	CAP160 protein
GLYMA16G23800	GLYMA16G23800.2	16	27666174	T	A	1053A>T	Glu351Asp	AT5G17680	disease resistance protein (TIR-NBS-LRR class), putative
GLYMA16G31331	GLYMA16G31331.1	16	34738002	C	A	1652C>A	Ala551Asp	AT5G52300	CAP160 protein

GLYMA16G31331	GLYMA16G31331.1	16	34738043	C	G	1693C>G	Leu565Val	AT5G52300	CAP160 protein
GLYMA16G31331	GLYMA16G31331.1	16	34738053	C	G	1703C>G	Ala568Gly	AT5G52300	CAP160 protein
GLYMA16G31331	GLYMA16G31331.1	16	34738056	A	T	1706A>T	Asp569Val	AT5G52300	CAP160 protein
GLYMA16G31445	GLYMA16G31445.1	16	34827935	C	A	1138C>A	Gln380Lys	AT5G01890	Leucine-rich receptor-like protein kinase family protein
GLYMA16G31445	GLYMA16G31445.1	16	34828119	T	C	1322T>C	Leu441Ser	AT5G01890	Leucine-rich receptor-like protein kinase family protein
GLYMA16G33616	GLYMA16G33616.1	16	36479733	G	C	43G>C	Asp15His	AT5G36930	Disease resistance protein (TIR-NBS-LRR class) family
GLYMA16G24920	GLYMA16G24920.2	16	28895500	C	T	1835G>A	Ser612Asn	AT5G17680	disease resistance protein (TIR-NBS-LRR class), putative
GLYMA16G24920	GLYMA16G24920.2	16	28895553	A	T	1782T>A	Asp594Glu	AT5G17680	disease resistance protein (TIR-NBS-LRR class), putative
GLYMA16G24920	GLYMA16G24920.2	16	28895572	C	G	1763G>C	Trp588Ser	AT5G17680	disease resistance protein (TIR-NBS-LRR class), putative
GLYMA16G24920	GLYMA16G24920.2	16	28895618	C	T	1717G>A	Glu573Lys	AT5G17680	disease resistance protein (TIR-NBS-LRR class), putative
GLYMA16G19790	GLYMA16G19790.2	16	22052029	C	A	481C>A	Leu161Ile	AT4G37990	elicitor-activated gene 3-2
GLYMA16G19790	GLYMA16G19790.2	16	22052078	T	C	530T>C	Ile177Thr	AT4G37990	elicitor-activated gene 3-2
GLYMA16G33616	GLYMA16G33616.1	16	36483791	C	G	3440C>G	Thr1147Arg	AT5G36930	Disease resistance protein (TIR-NBS-LRR class) family
GLYMA16G33616	GLYMA16G33616.1	16	36483794	A	G	3443A>G	Asn1148Ser	AT5G36930	Disease resistance protein (TIR-NBS-LRR class) family
GLYMA16G28460	GLYMA16G28460.2	16	32409863	A	G	1385A>G	Glu462Gly	AT2G15080	receptor like protein 19
GLYMA16G28460	GLYMA16G28460.2	16	32409890	T	C	1412T>C	Val471Ala	AT2G15080	receptor like protein 19
GLYMA16G28460	GLYMA16G28460.2	16	32410166	C	A	1688C>A	Thr563Asn	AT2G15080	receptor like protein 19
GLYMA16G28460	GLYMA16G28460.2	16	32410192	G	T	1714G>T	Val572Phe	AT2G15080	receptor like protein 19

GLYMA16G28460	GLYMA16G28460.2	16	32410613	C	G	2135C>G	Ser712Cys	AT2G15080	receptor like protein 19
GLYMA16G28460	GLYMA16G28460.2	16	32410795	G	T	2317G>T	Asp773Tyr	AT2G15080	receptor like protein 19
GLYMA16G33616	GLYMA16G33616.2	16	36479733	G	C	43G>C	Asp15His	AT5G36930	Disease resistance protein (TIR-NBS-LRR class) family
GLYMA16G28460	GLYMA16G28460.2	16	32410849	T	A	2371T>A	Phe791Ile	AT2G15080	receptor like protein 19
GLYMA16G28480	GLYMA16G28480.1	16	32421927	C	A	92C>A	Thr31Asn	AT2G15080	receptor like protein 19
GLYMA16G28480	GLYMA16G28480.1	16	32422017	C	G	182C>G	Thr61Arg	AT2G15080	receptor like protein 19
GLYMA16G28480	GLYMA16G28480.1	16	32422091	G	C	256G>C	Glu86Gln	AT2G15080	receptor like protein 19
GLYMA16G28480	GLYMA16G28480.1	16	32422093	G	C	258G>C	Glu86Asp	AT2G15080	receptor like protein 19
GLYMA16G28480	GLYMA16G28480.1	16	32422110	G	A	275G>A	Ser92Asn	AT2G15080	receptor like protein 19
GLYMA16G33616	GLYMA16G33616.2	16	36483791	C	G	3371C>G	Thr1124Arg	AT5G36930	Disease resistance protein (TIR-NBS-LRR class) family
GLYMA16G33616	GLYMA16G33616.2	16	36483794	A	G	3374A>G	Asn1125Ser	AT5G36930	Disease resistance protein (TIR-NBS-LRR class) family
GLYMA16G28480	GLYMA16G28480.1	16	32422112	C	G	277C>G	Arg93Gly	AT2G15080	receptor like protein 19
GLYMA16G28480	GLYMA16G28480.1	16	32422823	T	C	988T>C	Trp330Arg	AT2G15080	receptor like protein 19
GLYMA16G28480	GLYMA16G28480.1	16	32422838	T	C	1003T>C	Tyr335His	AT2G15080	receptor like protein 19
GLYMA16G28480	GLYMA16G28480.1	16	32423699	T	A	1864T>A	Leu622Met	AT2G15080	receptor like protein 19
GLYMA16G28520	GLYMA16G28520.2	16	32450649	A	G	257A>G	Asp86Gly	AT3G05650	receptor like protein 32
GLYMA16G28520	GLYMA16G28520.2	16	32451560	G	C	1168G>C	Glu390Gln	AT3G05650	receptor like protein 32
GLYMA0017S50	GLYMA0017S50.1	17	468	G	C	415C>G	Leu139Val	AT2G47300	ribonuclease Ps
GLYMA17G24236	GLYMA17G24236.1	17	24567350	A	C	943T>G	Tyr315Asp	AT5G33406	hAT dimerisation domain-containing protein / transposase-related
GLYMA0017S50	GLYMA0017S50.1	17	866	A	C	113T>G	Val38Gly	AT2G47300	ribonuclease Ps

GLYMA0017S50	GLYMA0017S50.1	17	870	T	C	109A>G	Arg37Gly	AT2G47300	ribonuclease Ps
GLYMA17G08060	GLYMA17G08060.2	17	5962415	A	G	575A>G	Gln192Arg	N/A	N/A
GLYMA17G08060	GLYMA17G08060.2	17	5962423	G	T	583G>T	Ala195Ser	N/A	N/A
GLYMA17G08060	GLYMA17G08060.2	17	5962439	C	G	599C>G	Thr200Ser	N/A	N/A
GLYMA17G24236	GLYMA17G24236.1	17	24568184	C	T	332G>A	Arg111His	AT5G33406	hAT dimerisation domain-containing protein / transposase-related
GLYMA17G24236	GLYMA17G24236.1	17	24568212	C	T	304G>A	Val102Ile	AT5G33406	hAT dimerisation domain-containing protein / transposase-related
GLYMA17G22453	GLYMA17G22453.1	17	22028487	G	T	180G>T	Gln60His	AT5G24910	cytochrome P450, family 714, subfamily A, polypeptide 1
GLYMA17G22453	GLYMA17G22453.1	17	22028515	A	G	208A>G	Asn70Asp	AT5G24910	cytochrome P450, family 714, subfamily A, polypeptide 1
GLYMA17G23790	GLYMA17G23790.1	17	23948534	T	G	419A>C	Tyr140Ser	ATMG01410	open reading frame 204
GLYMA17G23790	GLYMA17G23790.1	17	23948683	T	C	299A>G	Asn100Ser	ATMG01410	open reading frame 204
GLYMA17G23790	GLYMA17G23790.1	17	23948707	A	G	275T>C	Phe92Ser	ATMG01410	open reading frame 204
GLYMA17G23790	GLYMA17G23790.1	17	23948761	T	C	250A>G	Ile84Val	ATMG01410	open reading frame 204
GLYMA17G24236	GLYMA17G24236.1	17	24567052	G	C	1072C>G	Leu358Val	AT5G33406	hAT dimerisation domain-containing protein / transposase-related
GLYMA17G24236	GLYMA17G24236.1	17	24567070	A	C	1054T>G	Phe352Val	AT5G33406	hAT dimerisation domain-containing protein / transposase-related
GLYMA0017S50	GLYMA0017S50.1	17	497	G	C	386C>G	Thr129Ser	AT2G47300	ribonuclease Ps
GLYMA17G08060	GLYMA17G08060.2	17	5962758	A	T	834A>T	Glu278Asp	N/A	N/A
GLYMA17G08060	GLYMA17G08060.2	17	5962791	T	A	867T>A	His289Gln	N/A	N/A
GLYMA17G28555	GLYMA17G28555.1	17	30440981	G	A	347C>T	Pro116Leu	AT3G51690	PIF1 helicase
GLYMA17G28555	GLYMA17G28555.1	17	30441138	T	G	190A>C	Met64Leu	AT3G51690	PIF1 helicase

GLYMA17G28555	GLYMA17G28555.1	17	30441209	G	C	119C>G	Thr40Ser	AT3G51690	PIF1 helicase
GLYMA17G31651	GLYMA17G31651.1	17	34802692	G	A	464C>T	Thr155Ile	AT5G07050	nodulin MtN21 /EamA-like transporter family protein
GLYMA17G31651	GLYMA17G31651.1	17	34802704	G	A	452C>T	Pro151Leu	AT5G07050	nodulin MtN21 /EamA-like transporter family protein
GLYMA17G31651	GLYMA17G31651.1	17	34802705	G	A	451C>T	Pro151Ser	AT5G07050	nodulin MtN21 /EamA-like transporter family protein
GLYMA17G31651	GLYMA17G31651.1	17	34802718	C	T	438G>A	Met146Ile	AT5G07050	nodulin MtN21 /EamA-like transporter family protein
GLYMA17G32051	GLYMA17G32051.1	17	35269561	C	T	1151C>T	Ala384Val	AT4G32250	Protein kinase superfamily protein
GLYMA17G32051	GLYMA17G32051.1	17	35269873	G	A	1463G>A	Arg488His	AT4G32250	Protein kinase superfamily protein
GLYMA17G32051	GLYMA17G32051.2	17	35269561	C	T	1151C>T	Ala384Val	AT4G32250	Protein kinase superfamily protein
GLYMA17G32051	GLYMA17G32051.2	17	35269873	G	A	1463G>A	Arg488His	AT4G32250	Protein kinase superfamily protein
GLYMA17G32492	GLYMA17G32492.1	17	36118944	T	G	1500A>C	Lys500Asn	AT2G25290	N/A
GLYMA17G32492	GLYMA17G32492.1	17	36119027	A	G	1417T>C	Ser473Pro	AT2G25290	N/A
GLYMA17G32492	GLYMA17G32492.1	17	36119080	A	C	1364T>G	Leu455Arg	AT2G25290	N/A
GLYMA17G32492	GLYMA17G32492.1	17	36119098	A	G	1346T>C	Val449Ala	AT2G25290	N/A
GLYMA17G32492	GLYMA17G32492.1	17	36119390	T	C	1054A>G	Met352Val	AT2G25290	N/A
GLYMA17G32492	GLYMA17G32492.1	17	36120713	C	T	172G>A	Ala58Thr	AT2G25290	N/A
GLYMA18G01075	GLYMA18G01075.1	18	548117	G	A	4795G>A	Gly1599Ser	AT1G55540	Nuclear pore complex protein
GLYMA18G01075	GLYMA18G01075.1	18	548364	G	T	5042G>T	Gly1681Val	AT1G55540	Nuclear pore complex protein
GLYMA18G01075	GLYMA18G01075.2	18	548117	G	A	3547G>A	Gly1183Ser	AT1G55540	Nuclear pore complex protein
GLYMA18G01075	GLYMA18G01075.2	18	548364	G	T	3794G>T	Gly1265Val	AT1G55540	Nuclear pore complex protein
GLYMA18G08581	GLYMA18G08581.1	18	7305541	G	A	343G>A	Asp115Asn	AT3G51690	PIF1 helicase
GLYMA18G08581	GLYMA18G08581.1	18	7305568	T	G	370T>G	Ser124Ala	AT3G51690	PIF1 helicase
GLYMA18G08581	GLYMA18G08581.1	18	7305586	G	A	388G>A	Ala130Thr	AT3G51690	PIF1 helicase
GLYMA18G09314	GLYMA18G09314.1	18	8203335	G	T	338C>A	Thr113Asn	AT3G07040	NB-ARC domain-

									containing disease resistance protein
GLYMA18G09314	GLYMA18G09314.1	18	8203342	T	C	331A>G	Asn111Asp	AT3G07040	NB-ARC domain-containing disease resistance protein
GLYMA18G36216	GLYMA18G36216.1	18	42545154	G	A	10G>A	Glu4Lys	AT1G47790	F-box and associated interaction domains-containing protein
GLYMA18G09314	GLYMA18G09314.1	18	8203458	A	C	215T>G	Leu72Arg	AT3G07040	NB-ARC domain-containing disease resistance protein
GLYMA18G09314	GLYMA18G09314.1	18	8203482	G	T	191C>A	Ala64Asp	AT3G07040	NB-ARC domain-containing disease resistance protein
GLYMA18G36216	GLYMA18G36216.1	18	42545305	A	G	161A>G	Asn54Ser	AT1G47790	F-box and associated interaction domains-containing protein
GLYMA18G10625	GLYMA18G10625.1	18	9482979	T	C	1607T>C	Leu536Ser	AT3G07040	NB-ARC domain-containing disease resistance protein
GLYMA18G36401	GLYMA18G36401.1	18	42696448	C	T	790C>T	His264Tyr	AT3G23880	F-box and associated interaction domains-containing protein
GLYMA18G16414	GLYMA18G16414.1	18	17039576	T	C	122T>C	Phe41Ser	AT5G03610	GDSL-like Lipase/Acylhydrolase superfamily protein
GLYMA18G10625	GLYMA18G10625.1	18	9483049	G	T	1677G>T	Lys559Asn	AT3G07040	NB-ARC domain-containing disease resistance protein
GLYMA18G10625	GLYMA18G10625.1	18	9483074	T	C	1702T>C	Trp568Arg	AT3G07040	NB-ARC domain-containing disease resistance protein
GLYMA18G16414	GLYMA18G16414.1	18	17039561	G	T	107G>T	Gly36Val	AT5G03610	GDSL-like Lipase/Acylhydrolase superfamily protein
GLYMA18G48900	GLYMA18G48900.1	18	58321664	C	G	1702G>C	Ala568Pro	AT4G08850	Leucine-rich repeat receptor-like protein kinase family protein
GLYMA18G16414	GLYMA18G16414.1	18	17039675	T	A	221T>A	Val74Asp	AT5G03610	GDSL-like Lipase/Acylhydrolase superfamily protein
GLYMA18G18360	GLYMA18G18360.1	18	19858207	G	T	58G>T	Val20Leu	AT1G12000	Phosphofructokinase family protein

GLYMA18G18360	GLYMA18G18360.1	18	19858264	G	A	115G>A	Val39Met	AT1G12000	Phosphofructokinase family protein
GLYMA18G18360	GLYMA18G18360.1	18	19858271	G	A	122G>A	Arg41His	AT1G12000	Phosphofructokinase family protein
GLYMA18G18360	GLYMA18G18360.1	18	19858298	T	G	149T>G	Leu50Arg	AT1G12000	Phosphofructokinase family protein
GLYMA18G18360	GLYMA18G18360.1	18	19858399	A	G	250A>G	Ile84Val	AT1G12000	Phosphofructokinase family protein
GLYMA18G18360	GLYMA18G18360.1	18	19858465	C	T	316C>T	Arg106Cys	AT1G12000	Phosphofructokinase family protein
GLYMA18G18360	GLYMA18G18360.1	18	19858606	G	A	416G>A	Arg139His	AT1G12000	Phosphofructokinase family protein
GLYMA18G50510	GLYMA18G50510.1	18	59617352	G	A	1532C>T	Ala511Val	AT3G51550	Malectin/receptor-like protein kinase family protein
GLYMA18G18360	GLYMA18G18360.1	18	19858790	C	T	506C>T	Ala169Val	AT1G12000	Phosphofructokinase family protein
GLYMA18G18360	GLYMA18G18360.1	18	19858792	G	A	508G>A	Glu170Lys	AT1G12000	Phosphofructokinase family protein
GLYMA18G18360	GLYMA18G18360.1	18	19859324	C	T	656C>T	Ala219Val	AT1G12000	Phosphofructokinase family protein
GLYMA18G18360	GLYMA18G18360.1	18	19859357	G	A	689G>A	Gly230Asp	AT1G12000	Phosphofructokinase family protein
GLYMA18G18360	GLYMA18G18360.1	18	19859849	G	T	803G>T	Gly268Val	AT1G12000	Phosphofructokinase family protein
GLYMA18G32250	GLYMA18G32250.1	18	37331379	C	A	110C>A	Thr37Lys	ATCG01060	iron-sulfur cluster binding;electron carriers;4 iron, 4 sulfur cluster binding
GLYMA18G32250	GLYMA18G32250.1	18	37331391	T	C	122T>C	Phe41Ser	ATCG01060	iron-sulfur cluster binding;electron carriers;4 iron, 4 sulfur cluster binding
GLYMA18G32250	GLYMA18G32250.1	18	37331403	T	C	134T>C	Ile45Thr	ATCG01060	iron-sulfur cluster binding;electron carriers;4 iron, 4 sulfur cluster binding
GLYMA18G32744	GLYMA18G32744.1	18	38164223	G	A	348G>A	Met116Ile	AT5G41980	N/A
GLYMA18G32744	GLYMA18G32744.1	18	38164258	G	T	383G>T	Arg128Ile	AT5G41980	N/A
GLYMA18G32744	GLYMA18G32744.1	18	38164374	C	A	499C>A	Leu167Ile	AT5G41980	N/A
GLYMA18G32744	GLYMA18G32744.1	18	38164380	A	G	505A>G	Ser169Gly	AT5G41980	N/A

GLYMA18G32744	GLYMA18G32744.1	18	38164417	A	G	542A>G	Asn181Ser	AT5G41980	N/A
GLYMA18G35600	GLYMA18G35600.1	18	41926633	A	C	129A>C	Lys43Asn	AT5G06270	N/A
GLYMA18G35600	GLYMA18G35600.1	18	41926640	G	A	136G>A	Gly46Ser	AT5G06270	N/A
GLYMA18G35790	GLYMA18G35790.1	18	42063998	T	C	578A>G	Asp193Gly	AT1G65450	HXXXD-type acyl-transferase family protein
GLYMA18G35790	GLYMA18G35790.1	18	42064071	C	T	505G>A	Ala169Thr	AT1G65450	HXXXD-type acyl-transferase family protein
GLYMA18G35790	GLYMA18G35790.1	18	42064088	C	A	488G>T	Cys163Phe	AT1G65450	HXXXD-type acyl-transferase family protein
GLYMA18G35790	GLYMA18G35790.1	18	42065090	C	T	5G>A	Gly2Asp	AT1G65450	HXXXD-type acyl-transferase family protein
GLYMA18G50510	GLYMA18G50510.1	18	59618078	A	T	806T>A	Leu269Gln	AT3G51550	Malectin/receptor-like protein kinase family protein
GLYMA18G36216	GLYMA18G36216.1	18	42545163	T	C	19T>C	Ser7Pro	AT1G47790	F-box and associated interaction domains-containing protein
GLYMA18G36216	GLYMA18G36216.1	18	42545168	T	G	24T>G	Cys8Trp	AT1G47790	F-box and associated interaction domains-containing protein
GLYMA18G36216	GLYMA18G36216.1	18	42545187	G	A	43G>A	Glu15Lys	AT1G47790	F-box and associated interaction domains-containing protein
GLYMA18G36216	GLYMA18G36216.1	18	42545251	A	G	107A>G	Tyr36Cys	AT1G47790	F-box and associated interaction domains-containing protein
GLYMA18G50510	GLYMA18G50510.1	18	59618408	C	G	476G>C	Cys159Ser	AT3G51550	Malectin/receptor-like protein kinase family protein
GLYMA18G36216	GLYMA18G36216.1	18	42545391	T	A	247T>A	Ser83Thr	AT1G47790	F-box and associated interaction domains-containing protein
GLYMA18G36401	GLYMA18G36401.1	18	42696020	G	C	415G>C	Gly139Arg	AT3G23880	F-box and associated interaction domains-containing protein
GLYMA18G36401	GLYMA18G36401.1	18	42696062	G	T	457G>T	Ala153Ser	AT3G23880	F-box and associated interaction domains-containing protein

GLYMA18G36401	GLYMA18G36401.1	18	42696129	G	C	524G>C	Ser175Thr	AT3G23880	F-box and associated interaction domains-containing protein
GLYMA18G50510	GLYMA18G50510.1	18	59618620	C	G	264G>C	Leu88Phe	AT3G51550	Malectin/receptor-like protein kinase family protein
GLYMA18G36401	GLYMA18G36401.1	18	42696460	C	T	802C>T	Arg268Cys	AT3G23880	F-box and associated interaction domains-containing protein
GLYMA18G36401	GLYMA18G36401.1	18	42696547	G	A	889G>A	Asp297Asn	AT3G23880	F-box and associated interaction domains-containing protein
GLYMA18G37410	GLYMA18G37410.1	18	44679557	C	T	265C>T	Arg89Cys	N/A	N/A
GLYMA18G37410	GLYMA18G37410.1	18	44680907	G	A	589G>A	Glu197Lys	N/A	N/A
GLYMA18G37410	GLYMA18G37410.1	18	44681574	A	G	1058A>G	Glu353Gly	N/A	N/A
GLYMA18G37410	GLYMA18G37410.1	18	44681594	A	G	1078A>G	Ile360Val	N/A	N/A
GLYMA18G37410	GLYMA18G37410.1	18	44681606	A	G	1090A>G	Thr364Ala	N/A	N/A
GLYMA18G37410	GLYMA18G37410.1	18	44682095	C	T	1415C>T	Pro472Leu	N/A	N/A
GLYMA18G37410	GLYMA18G37410.1	18	44682119	G	A	1439G>A	Arg480Lys	N/A	N/A
GLYMA18G37620	GLYMA18G37620.2	18	44872858	C	T	328G>A	Gly110Arg	AT1G74410	RING/U-box superfamily protein
GLYMA18G37620	GLYMA18G37620.2	18	44872860	T	C	326A>G	Gln109Arg	AT1G74410	RING/U-box superfamily protein
GLYMA18G50510	GLYMA18G50510.1	18	59618690	T	C	194A>G	Asn65Ser	AT3G51550	Malectin/receptor-like protein kinase family protein
GLYMA18G37620	GLYMA18G37620.2	18	44873271	T	C	130A>G	Ile44Val	AT1G74410	RING/U-box superfamily protein
GLYMA18G39181	GLYMA18G39181.1	18	47135066	C	G	155G>C	Arg52Pro	AT4G38180	FAR1-related sequence 5
GLYMA18G09314	GLYMA18G09314.1	18	8203389	G	C	284C>G	Thr95Ser	AT3G07040	NB-ARC domain-containing disease resistance protein
GLYMA18G40870	GLYMA18G40870.1	18	49604663	G	A	227C>T	Ala76Val	AT1G53903	Protein of unknown function (DUF581)
GLYMA18G40870	GLYMA18G40870.1	18	49604681	T	A	209A>T	Glu70Val	AT1G53903	Protein of unknown function (DUF581)
GLYMA18G40870	GLYMA18G40870.1	18	49604697	C	T	193G>A	Gly65Ser	AT1G53903	Protein of unknown function (DUF581)
GLYMA18G42160	GLYMA18G42160.3	18	51122285	T	A	350T>A	Leu117His	AT3G09110	Protein of unknown function (DUF674)

GLYMA18G42160	GLYMA18G42160.3	18	51122522	T	C	587T>C	Val196Ala	AT3G09110	Protein of unknown function (DUF674)
GLYMA18G42160	GLYMA18G42160.3	18	51122814	A	C	784A>C	Met262Leu	AT3G09110	Protein of unknown function (DUF674)
GLYMA18G09314	GLYMA18G09314.1	18	8203509	C	T	164G>A	Cys55Tyr	AT3G07040	NB-ARC domain-containing disease resistance protein
GLYMA18G46026	GLYMA18G46026.1	18	55754062	A	T	755A>T	Lys252Met	AT4G27190	NB-ARC domain-containing disease resistance protein
GLYMA18G10625	GLYMA18G10625.1	18	9482992	G	T	1620G>T	Met540Ile	AT3G07040	NB-ARC domain-containing disease resistance protein
GLYMA18G10625	GLYMA18G10625.1	18	9483001	C	G	1629C>G	Asn543Lys	AT3G07040	NB-ARC domain-containing disease resistance protein
GLYMA18G46026	GLYMA18G46026.1	18	55754077	A	G	770A>G	Asn257Ser	AT4G27190	NB-ARC domain-containing disease resistance protein
GLYMA18G46026	GLYMA18G46026.1	18	55760673	A	T	4603A>T	Asn1535Tyr	AT4G27190	NB-ARC domain-containing disease resistance protein
GLYMA18G46050	GLYMA18G46050.5	18	55811032	C	G	4886C>G	Ala1629Gly	AT4G27220	NB-ARC domain-containing disease resistance protein
GLYMA18G46050	GLYMA18G46050.5	18	55811317	C	T	5171C>T	Thr1724Ile	AT4G27220	NB-ARC domain-containing disease resistance protein
GLYMA18G46050	GLYMA18G46050.5	18	55811365	C	G	5219C>G	Thr1740Ser	AT4G27220	NB-ARC domain-containing disease resistance protein
GLYMA18G46050	GLYMA18G46050.5	18	55817415	A	T	7862A>T	Gln2621Leu	AT4G27220	NB-ARC domain-containing disease resistance protein
GLYMA18G46050	GLYMA18G46050.5	18	55817423	A	G	7870A>G	Lys2624Glu	AT4G27220	NB-ARC domain-containing disease resistance protein
GLYMA18G46050	GLYMA18G46050.5	18	55817449	T	G	7896T>G	Asp2632Glu	AT4G27220	NB-ARC domain-containing disease resistance protein
GLYMA18G46050	GLYMA18G46050.5	18	55817531	G	C	7978G>C	Glu2660Gln	AT4G27220	NB-ARC domain-containing disease resistance protein

GLYMA18G46050	GLYMA18G46050.5	18	55811374	G	C	5228G>C	Arg1743Thr	AT4G27220	NB-ARC domain-containing disease resistance protein
GLYMA18G46050	GLYMA18G46050.5	18	55812344	C	G	6087C>G	Ser2029Arg	AT4G27220	NB-ARC domain-containing disease resistance protein
GLYMA18G46080	GLYMA18G46080.1	18	55842716	C	A	1082G>T	Arg361Leu	AT5G01150	Protein of unknown function (DUF674)
GLYMA18G18360	GLYMA18G18360.1	18	19858650	C	T	460C>T	Arg154Trp	AT1G12000	Phosphofructokinase family protein
GLYMA18G46080	GLYMA18G46080.1	18	55842779	G	A	1019C>T	Ala340Val	AT5G01150	Protein of unknown function (DUF674)
GLYMA18G40870	GLYMA18G40870.1	18	49604582	G	T	308C>A	Ser103Tyr	AT1G53903	Protein of unknown function (DUF581)
GLYMA18G48900	GLYMA18G48900.1	18	58321784	C	G	1582G>C	Glu528Gln	AT4G08850	Leucine-rich repeat receptor-like protein kinase family protein
GLYMA18G48900	GLYMA18G48900.1	18	58323061	C	T	305G>A	Gly102Glu	AT4G08850	Leucine-rich repeat receptor-like protein kinase family protein
GLYMA18G48900	GLYMA18G48900.1	18	58323315	C	G	51G>C	Glu17Asp	AT4G08850	Leucine-rich repeat receptor-like protein kinase family protein
GLYMA18G42160	GLYMA18G42160.3	18	51122946	A	T	916A>T	Ser306Cys	AT3G09110	Protein of unknown function (DUF674)
GLYMA18G50510	GLYMA18G50510.1	18	59617817	T	A	1067A>T	Lys356Met	AT3G51550	Malectin/receptor-like protein kinase family protein
GLYMA18G46080	GLYMA18G46080.1	18	55842118	T	C	1523A>G	Lys508Arg	AT5G01150	Protein of unknown function (DUF674)
GLYMA18G50510	GLYMA18G50510.1	18	59618157	C	G	727G>C	Glu243Gln	AT3G51550	Malectin/receptor-like protein kinase family protein
GLYMA18G46080	GLYMA18G46080.1	18	55842489	C	T	1309G>A	Asp437Asn	AT5G01150	Protein of unknown function (DUF674)
GLYMA18G46080	GLYMA18G46080.1	18	55842737	A	G	1061T>C	Ile354Thr	AT5G01150	Protein of unknown function (DUF674)
GLYMA18G37620	GLYMA18G37620.2	18	44873259	T	C	142A>G	Thr48Ala	AT1G74410	RING/U-box superfamily protein
GLYMA19G06650	GLYMA19G06650.1	19	7748057	G	C	1031C>G	Thr344Arg	AT3G23880	F-box and associated interaction domains-containing protein
GLYMA19G31870	GLYMA19G31870.1	19	39644345	C	A	237G>T	Arg79Ser	AT4G20990	alpha carbonic anhydrase 4

GLYMA19G07557	GLYMA19G07557.1	19	8992048	C	A	169G>T	Val57Leu	ATCG00150	ATPase, F0 complex, subunit A protein
GLYMA19G06890	GLYMA19G06890.1	19	8082050	C	T	731C>T	Ala244Val	AT5G33370	GDSL-like Lipase/Acylhydrolase superfamily protein
GLYMA19G06890	GLYMA19G06890.1	19	8082187	G	C	868G>C	Ala290Pro	AT5G33370	GDSL-like Lipase/Acylhydrolase superfamily protein
GLYMA19G07557	GLYMA19G07557.1	19	8991939	T	G	278A>C	Asp93Ala	ATCG00150	ATPase, F0 complex, subunit A protein
GLYMA19G07557	GLYMA19G07557.1	19	8992068	T	C	149A>G	Lys50Arg	ATCG00150	ATPase, F0 complex, subunit A protein
GLYMA19G07557	GLYMA19G07557.1	19	8992069	T	C	148A>G	Lys50Glu	ATCG00150	ATPase, F0 complex, subunit A protein
GLYMA19G10040	GLYMA19G10040.1	19	11965160	C	T	158C>T	Ala53Val	AT3G03740	BTB-POZ and MATH domain 4
GLYMA19G10040	GLYMA19G10040.1	19	11965169	C	T	167C>T	Thr56Ile	AT3G03740	BTB-POZ and MATH domain 4
GLYMA19G10040	GLYMA19G10040.1	19	11965184	G	A	182G>A	Gly61Glu	AT3G03740	BTB-POZ and MATH domain 4
GLYMA19G10040	GLYMA19G10040.1	19	11965211	C	T	209C>T	Pro70Leu	AT3G03740	BTB-POZ and MATH domain 4
GLYMA19G10040	GLYMA19G10040.1	19	11966407	A	C	914A>C	Tyr305Ser	AT3G03740	BTB-POZ and MATH domain 4
GLYMA19G10040	GLYMA19G10040.1	19	11965261	A	G	259A>G	Thr87Ala	AT3G03740	BTB-POZ and MATH domain 4
GLYMA19G10040	GLYMA19G10040.1	19	11965273	G	A	271G>A	Glu91Lys	AT3G03740	BTB-POZ and MATH domain 4
GLYMA19G10040	GLYMA19G10040.1	19	11966445	A	T	952A>T	Asn318Tyr	AT3G03740	BTB-POZ and MATH domain 4
GLYMA19G06650	GLYMA19G06650.1	19	7748331	C	T	757G>A	Val253Ile	AT3G23880	F-box and associated interaction domains-containing protein
GLYMA19G14700	GLYMA19G14700.2	19	17582005	G	A	1481G>A	Arg494His	AT1G11950	Transcription factor jumonji (jmjC) domain-containing protein
GLYMA19G14700	GLYMA19G14700.2	19	17583976	A	G	1915A>G	Ile639Val	AT1G11950	Transcription factor jumonji (jmjC) domain-containing protein
GLYMA19G14700	GLYMA19G14700.2	19	17584049	G	A	1988G>A	Gly663Glu	AT1G11950	Transcription factor jumonji (jmjC) domain-containing

									protein
GLYMA19G14700	GLYMA19G14700.2	19	17584183	C	G	2122C>G	Arg708Gly	AT1G11950	Transcription factor jumonji (jmjC) domain-containing protein
GLYMA19G14700	GLYMA19G14700.2	19	17584324	C	T	2263C>T	Arg755Trp	AT1G11950	Transcription factor jumonji (jmjC) domain-containing protein
GLYMA19G27052	GLYMA19G27052.1	19	34222711	A	G	79A>G	Ile27Val	AT3G51690	PIF1 helicase
GLYMA19G27052	GLYMA19G27052.1	19	34222768	G	A	136G>A	Ala46Thr	AT3G51690	PIF1 helicase
GLYMA19G27052	GLYMA19G27052.1	19	34222907	G	A	275G>A	Gly92Glu	AT3G51690	PIF1 helicase
GLYMA19G27052	GLYMA19G27052.1	19	34222914	G	C	282G>C	Met94Ile	AT3G51690	PIF1 helicase
GLYMA19G06890	GLYMA19G06890.1	19	8081070	G	C	203G>C	Ser68Thr	AT5G33370	GDSL-like Lipase/Acylhydrolase superfamily protein
GLYMA19G31651	GLYMA19G31651.1	19	39452431	C	T	431C>T	Thr144Ile	AT3G14460	LRR and NB-ARC domains-containing disease resistance protein
GLYMA19G31651	GLYMA19G31651.1	19	39452160	A	C	160A>C	Lys54Gln	AT3G14460	LRR and NB-ARC domains-containing disease resistance protein
GLYMA19G31651	GLYMA19G31651.1	19	39452776	C	G	776C>G	Pro259Arg	AT3G14460	LRR and NB-ARC domains-containing disease resistance protein
GLYMA19G31651	GLYMA19G31651.1	19	39452790	A	G	790A>G	Asn264Asp	AT3G14460	LRR and NB-ARC domains-containing disease resistance protein
GLYMA19G31651	GLYMA19G31651.1	19	39452844	C	A	844C>A	Gln282Lys	AT3G14460	LRR and NB-ARC domains-containing disease resistance protein
GLYMA19G31881	GLYMA19G31881.1	19	39649486	T	C	1349A>G	His450Arg	AT3G14460	LRR and NB-ARC domains-containing disease resistance protein
GLYMA19G31651	GLYMA19G31651.1	19	39452880	G	A	880G>A	Asp294Asn	AT3G14460	LRR and NB-ARC domains-containing

									disease resistance protein
GLYMA19G31870	GLYMA19G31870.1	19	39643445	A	G	427T>C	Tyr143His	AT4G20990	alpha carbonic anhydrase 4
GLYMA19G27052	GLYMA19G27052.1	19	34223270	A	G	557A>G	Glu186Gly	AT3G51690	PIF1 helicase
GLYMA19G31870	GLYMA19G31870.1	19	39644478	T	G	104A>C	Gln35Pro	AT4G20990	alpha carbonic anhydrase 4
GLYMA19G31881	GLYMA19G31881.1	19	39648308	G	C	2527C>G	Gln843Glu	AT3G14460	LRR and NB-ARC domains-containing disease resistance protein
GLYMA19G31881	GLYMA19G31881.1	19	39648317	T	C	2518A>G	Lys840Glu	AT3G14460	LRR and NB-ARC domains-containing disease resistance protein
GLYMA19G31881	GLYMA19G31881.1	19	39649154	T	A	1681A>T	Ile561Phe	AT3G14460	LRR and NB-ARC domains-containing disease resistance protein
GLYMA19G31881	GLYMA19G31881.1	19	39649336	T	C	1499A>G	Tyr500Cys	AT3G14460	LRR and NB-ARC domains-containing disease resistance protein
GLYMA19G31881	GLYMA19G31881.1	19	39649415	C	T	1420G>A	Glu474Lys	AT3G14460	LRR and NB-ARC domains-containing disease resistance protein
GLYMA19G27052	GLYMA19G27052.1	19	34223279	T	C	566T>C	Phe189Ser	AT3G51690	PIF1 helicase
GLYMA19G31881	GLYMA19G31881.1	19	39649658	A	C	1177T>G	Ser393Ala	AT3G14460	LRR and NB-ARC domains-containing disease resistance protein
GLYMA1057S00200	GLYMA1057S00200.1	scaffold_1057	3469	T	C	165A>G	Ile55Met	AT2G45550	cytochrome P450, family 76, subfamily C, polypeptide 4
GLYMA1057S00200	GLYMA1057S00200.1	scaffold_1057	3558	C	T	76G>A	Gly26Ser	AT2G45550	cytochrome P450, family 76, subfamily C, polypeptide 4
GLYMA1057S00200	GLYMA1057S00200.1	scaffold_1057	3567	T	C	67A>G	Arg23Gly	AT2G45550	cytochrome P450, family 76, subfamily C, polypeptide 4

NB-ARC, nucleotide-binding APAF-1 R proteins and CED-4; TIR-NBS-LRR, toll interleukin 1 receptor nucleotide-binding site leucine-rich repeat resistance proteins; N/A, not available.
