# Supplementary material for: Analysis of genome variants in dwarf soybean lines obtained in F6 derived from cross of normal parents (cultivated and wild soybean)
Source: Genomics Inform. 2021 Jun 30;19(2):e19. doi: 10.5808/gi.21024 (PMC8261272; doi:10.5808/gi.21024)
Supplement: Supplemental Table 3. — Distribution of missense SNP when compared with reference genome [file gi-21024suppl3.pdf]

Supplementary Table 3. Distribution of missense SNP when compared with reference genome

Chromosome No.	No. of genes with different genotypes	1282		1303		1282 + 1303	
		Loci with SNPs	Loci with homogeneous in dwarf[a]	Loci with SNPs	Loci with homogeneous in dwarf[a]	Loci with SNPs	Loci with homogeneous in dwarf[a]
1	9	17	8	29	24	5	3
2	12	20	8	56	39	5	0
3	6	7	5	10	4	0	0
4	5	51	11	24	6	16	2
5	2	19	0	15	14	1	0
6	7	15	11	9	3	1	0
7	11	58	35	63	40	14	8
8	6	34	13	16	10	7	2
9	5	11	3	10	4	3	0
10	5	17	15	8	3	2	2
11	3	4	3	6	5	1	1
12	1	1	0	2	2	0	0
13	9	28	18	27	8	4	3
14	2	3	2	6	4	1	1
15	6	22	13	41	5	6	1
16	16	74	51	67	33	10	7
17	9	18	8	18	8	3	0
18	22	49	14	57	29	13	2
19	9	27	7	20	13	5	1
20	1	3	1	1	0	0	0
Total	146	478	226	485	254	97	33

SNP, single nuclotide polymorphism.
