# Supplementary material for: Analysis of genome variants in dwarf soybean lines obtained in F6 derived from cross of normal parents (cultivated and wild soybean)
Source: Genomics Inform. 2021 Jun 30;19(2):e19. doi: 10.5808/gi.21024 (PMC8261272; doi:10.5808/gi.21024)
Supplement: Supplemental Table 4. — Nonsense and frameshift in both samples as compared to reference genome [file gi-21024suppl4.pdf]

Supplementary Table 4. Nonsense and frameshift in both samples as compared to reference genome

Non sense

gene	chrom	pos	ref	alt	dna	prot	effect	ygous in dv	TAIR TOP hit	TAIR TOP hit function
GLYMA02G02441	2	1834062	G	A	16C>T	Arg6*	Arg6*	No	AT1G14410	ssDNA-binding transcriptional regulator
GLYMA13G06151	13	6454142	C	T	566G>A	Trp189*	Trp189*	No	AT3G02100	UDP-Glycosyltransferase superfamily protein
GLYMA0086S00250	20	40432	G	A	1171C>T	Gln391*	Gln391*	Yes	AT3G51690	PIF1 helicase

Frameshift

gene	chrom	pos	ref	alt	dna	prot	effect	ygous in dv	TAIR TOP hit	TAIR TOP hit function
GLYMA02G07895	2	6231825	AATATATATAT	A,AATATATATATATAT	161_162insATAT	Tyr58fs	Tyr58fs	No	AT1G20380	Prolyl oligopeptidase family protein
GLYMA03G37750	3	44271256	TGGGGG	T,TGGGGGG	2037dupG	Ile680fs	Ile680fs	No	AT4G02070	MUTS homolog 6
GLYMA03G37750	3	44271322	G	GT,GTT	2054_2055dupTT	Ile686fs	Ile686fs	No	AT4G02070	MUTS homolog 6
GLYMA08G09520	8	6788920	T	TTTTTAATGATA	110_111insTATCA	Met38fs	Met38fs	No	AT4G20150	unknown protein
GLYMA08G26950	8	21285028	T	TG	71dupC	Thr25fs	Thr25fs	No	AT4G00660	RNAhelicase-like 8
GLYMA11G37110	11	38337736	TA	T	32delT	Leu11fs	Leu11fs	No	AT1G13710	cytochrome P450, family 78, subfamily A, polypeptide 5
GLYMA16G28670	16	32614346	AG	A	1807delG	Glu603fs	Glu603fs	No	AT2G34930	disease resistance family protein / LRR family protein
GLYMA16G28670	16	32614350	G	GT	1811dupT	Leu604fs	Leu604fs	No	AT2G34930	disease resistance family protein / LRR family protein
GLYMA18G32241	18	37330782	AGT	A	116_117delGT	Ser39fs	Ser39fs	No	ATCG01070	NADH-ubiquinone/plastoquinone oxidoreductase chain 4L
GLYMA18G32241	18	37330786	T	TGC	119_120insGC	Ile40fs	Ile40fs	No	ATCG01070	NADH-ubiquinone/plastoquinone oxidoreductase chain 4L
GLYMA18G46080	18	55842110	A	AT	1530dupA	Ter511fs	Ter511fs	No	AT5G01150	Protein of unknown function (DUF674)
GLYMA18G46080	18	55842124	C	CTA	1516_1517insTA	Ser506fs	Ser506fs	No	AT5G01150	Protein of unknown function (DUF674)
GLYMA18G46080	18	55842491	TCCAACAACCGG	T	1269_1306delCA	Ser423fs	Ser423fs	No	AT5G01150	Protein of unknown function (DUF674)
GLYMA18G48950	18	58356454	ACCCGGTG	A	239_245delCACC	Thr80fs	Thr80fs	No	AT4G08850	Leucine-rich repeat receptor-like protein kinase family protein
GLYMA18G48950	18	58356456	CCGGTGTAT	C	236_243delATAC	Tyr79fs	Tyr79fs	No	AT4G08850	Leucine-rich repeat receptor-like protein kinase family protein
GLYMA18G48950	18	58356463	AT	A	236delA	Tyr79fs	Tyr79fs	No	AT4G08850	Leucine-rich repeat receptor-like protein kinase family protein
GLYMA18G48950	18	58356466	AC	A	233delG	Cys78fs	Cys78fs	No	AT4G08850	Leucine-rich repeat receptor-like protein kinase family protein
GLYMA18G48950	18	58356469	TG	T	230delC	Pro77fs	Pro77fs	No	AT4G08850	Leucine-rich repeat receptor-like protein kinase family protein
GLYMA18G48950	18	58356481	GA	G	218delT	Val73fs	Val73fs	No	AT4G08850	Leucine-rich repeat receptor-like protein kinase family protein
GLYMA18G48950	18	58356485	G	GCCAA	214_215insTTGG	Thr72fs	Thr72fs	No	AT4G08850	Leucine-rich repeat receptor-like protein kinase family protein
